# DNA methylation markers in esophageal cancer

**DOI:** 10.3389/fgene.2024.1354195

**Published:** 2024-05-07

**Authors:** Yongle Xu, Zhenzhen Wang, Bing Pei, Jie Wang, Ying Xue, Guodong Zhao

**Affiliations:** ^1^ Suzhou Municipal Hospital, Gusu School, The Affiliated Suzhou Hospital of Nanjing Medical University, Nanjing Medical University, Suzhou, China; ^2^ Department of Laboratory Medicine, Affiliated Xuzhou Maternity and Child Healthcare Hospital of Xuzhou Medical University, Xuzhou, China; ^3^ Department of Clinical Laboratory, The Affiliated Suqian First People’s Hospital of Nanjing Medical University, Suqian, China; ^4^ Department of Spleen and Stomach Diseases, Kunshan Hospital of Traditional Chinese Medicine, Kunshan, China; ^5^ Zhejiang University of Technology, Hangzhou, China; ^6^ ZJUT Yinhu Research Institute of Innovation and Entrepreneurship, Hangzhou, China

**Keywords:** esophageal cancer, DNA methylation, cell free DNA, esophageal exfoliated cells, early detection

## Abstract

**Background:**

Esophageal cancer (EC) is a prevalent malignancy characterized by a low 5-year survival rate, primarily attributed to delayed diagnosis and limited therapeutic options. Currently, early detection of EC heavily relies on endoscopy and pathological examination, which pose challenges due to their invasiveness and high costs, leading to low patient compliance. The detection of DNA methylation offers a non-endoscopic, cost-effective, and secure approach that holds promising prospects for early EC detection.

**Methods:**

To identify improved methylation markers for early EC detection, we conducted a comprehensive review of relevant literature, summarized the performance of DNA methylation markers based on different input samples and analytical methods in EC early detection and screening.

**Findings:**

This review reveals that blood cell free DNA methylation-based method is an effective non-invasive method for early detection of EC, although there is still a need to improve its sensitivity and specificity. Another highly sensitive and specific non-endoscopic approach for early detection of EC is the esophageal exfoliated cells based-DNA methylation analysis. However, while there are substantial studies in esophageal adenocarcinoma, further more validation is required in esophageal squamous cell carcinoma.

**Conclusion:**

In conclusion, DNA methylation detection holds significant potential as an early detection and screening technology for EC.

## 1 Introduction

Esophageal cancer (EC) is a highly aggressive malignancy that arises from the esophageal epithelium (Talukdar et al., 2018). In 2020, it accounted for 604,100 new cases and resulted in 544,076 deaths worldwide, as reported by global epidemiological data (Sung et al., 2021). Although the incidence rate of EC ranks seventh and has shown a decline over the years, it remains a significant concern due to its exceptionally low 10% survival rate. Therefore, it is crucial to address this disease with utmost seriousness (Siegel et al., 2021).

EC encompasses two main histological types: esophageal squamous cell carcinoma (ESCC) and esophageal adenocarcinoma (EAC) (Rogers and Ajani, 2022). ESCC, originating from the squamous epithelial cells lining the esophagus, accounts for approximately 90% of EC cases worldwide, making it the most prevalent subtype, particularly in Asia, including China, Iran, and other parts of Central Asia (Henry et al., 2014). On the other hand, EAC represents around 10% of all EC and is more commonly observed in Western countries such as the United States, Canada, Australia, and Western Europe (Zheng et al., 2019). The development of these malignancies is typically a gradual process, spanning from normal tissue to cancer formation. In the case of ESCC, the most common precursor lesion is squamous dysplasia, characterized by the presence of abnormal cells in the squamous epithelium lining the esophagus. Squamous dysplasia can be categorized as low-grade dysplasia (LGD), high-grade dysplasia (HGD), or carcinoma *in situ* (CIS) (Akiyama et al., 2014). Conversely, in the context of EAC, the precursor lesion is known as Barrett’s esophagus (BE), a condition in which the normal squamous epithelium of the esophagus is replaced by columnar cells, often resulting from chronic gastroesophageal reflux disease (GERD). BE can progress from LGD to HGD and ultimately to invasive adenocarcinoma (Shaheen et al., 2016). Although ESCC and EAC follow distinct tumorigenic pathways, a common challenge lies in the difficulty of screening for EC when mucosal changes cannot be visualized readily by endoscopy. Consequently, the identification of EC in precancerous lesions is crucial for improving patient outcomes and reducing mortality rates.

Barium swallow, a type of X-ray imaging, employs a contrast dye to enhance the visibility of the esophagus, enabling the detection of any anomalies in its lining (Levine and Rubesin, 2017). However, this method is limited in its ability to detect subtle changes in the esophageal wall, including precancerous lesions. Presently, endoscopy stands as the primary screening approach for EC. This procedure involves the insertion of a flexible, slender tube with a camera at its tip into the esophagus, enabling the identification of any irregularities. Additionally, during an endoscopy, a physician can obtain a small tissue sample (biopsy) from suspicious areas within the esophagus, which can be further examined under a microscope to identify cancerous signs. This method is considered the most accurate for early EC diagnosis (Buxbaum and Eloubeidi, 2009). Nonetheless, due to its invasive nature, dietary restrictions, and high costs, its compliance rate remains low (Evans et al., 2013).

DNA methylation is a prominent epigenetic modification process in which cytosine is transformed into 5-methylcytosine (5-mC) through the catalytic action of DNA methyltransferase (Dnmt) utilizing S-adenosylmethionine as a methyl donor (Siegfried and Simon, 2010). Although it does not alter the primary structure of DNA, DNA methylation plays a crucial role in cellular development, gene expression, and genome stability (van Eijk et al., 2012). CpG island hypermethylation is a frequently observed phenomenon in tumors and serves as the third mechanism, alongside mutation and deletion, for the inactivation of tumor suppressor genes (Irizarry et al., 2009a; Irizarry et al., 2009b). Notably, DNA methylation can be detected in various bodily fluids such as blood, stool, urine, and cerebrospinal fluid (Liu et al., 2020; Rahat et al., 2020). This characteristic, combined with its superior stability, sensitivity, and specificity compared to other cell-free nucleic acid markers (such as miRNA, lncRNA, or mRNA), positions DNA methylation as a promising non-invasive marker for early cancer detection (Jamshidi et al., 2022).

Over the past decade, there has been a growing interest in detecting DNA methylation in esophageal exfoliated cells and blood (Reeh et al., 2015; Moinova et al., 2018; Wang et al., 2019; Prasoppokakorn et al., 2022). A novel diagnostic technique known as esophageal balloon cytology detection has emerged, which combines a non-endoscopic cytologic sampling device with an immunohistochemical biomarker. This method enables the collection of exfoliated cells by having the patient swallow a specially designed gelatin capsule that expands in the esophagus (Lao-Sirieix et al., 2009; Kadri et al., 2010). This technology not only allows for the observation of cell morphology but also facilitates the detection of cancer biomarkers, including DNA methylation biomarkers (Codipilly et al., 2018). Circulating cell-free DNA (cfDNA) released from primary tumors or metastases has garnered significant attention, with multiple studies confirming its higher abundance in cancer patients compared to healthy individuals (Husain et al., 2017; Váraljai et al., 2020; Schlick et al., 2021). Detection of cfDNA methylation can be performed in various body fluids such as urine (Husain et al., 2017), saliva (Wang et al., 2015), cerebrospinal (De Mattos-Arruda et al., 2015), offering a non-invasive approach that holds great potential as a screening technology for malignancies.

For the whole process of the DNA methylation analysis, the input sample types and analytical methods, include the DNA isolation, conversion and detection, are the key factors will affect the performance of marker discovery and application. Therefore, the aim of this review is to synthesize findings from diverse studies, evaluate the performance of DNA methylation marker in different sample types and methods for EC early detection and screening, and discuss the prospects and challenges associated with their future application.

A literature search was performed on PubMed, Medline and Web of Science databases until December 2023 using the following key words query: a) DNA methylation OR methylation marker OR methylation biomarker OR methylation panel; b) (and) Esophageal cancer OR esophageal squamous cell carcinoma OR esophageal adenocarcinoma OR barrett’s esophagus; c) (and) Detection OR diagnosis OR screening. Some studies were excluded if they were a) The focus is on treatment and prognosis of EC; b) Animal studies; c) Studies that did not specify sensitivity or specificity of the markers. We extracted data from every manuscript were as follows: publication year, sample types, sample size, DNA isolation and conversion method, analytical method, sensitivity and specificity of detecting and its AUCs, which formed the tables in this review, to show a comprehensive and detailed comparison.

In this review, 50 relevant articles were included for analysis of DNA methylation markers in early detection of EC (Supplementary Table S1), these studies examined various sample types including tissue, blood, and esophageal exfoliated cells, using different methods such as methylation-specific PCR (MSP), quantitative methylation-specific PCR (qMSP), droplet digital PCR (ddPCR), among others. Consequently, we summarized and extracted the performance characteristics of the markers based on the respective sample types. All the studies included in this review utilized a gene-specific approach to evaluate the methylation status of 65 genes in relation to EC and its premalignant lesions, including BE, HGD, and LGD. These genes were assessed either individually or as part of a panel. Some genes were reported multiple times, while others were mentioned only once. Among the genes evaluated multiple times as individual methylation markers were *SFRP1*, *TAC1*, *PAX1*, *ZNF582*, and *ZNF569*. On the other hand, *P16*, *RAR*, *MGMT*, *RASSF1A*, *TFPI2* and *ELMO1* were frequently included in panels. It is worth noting that the performance exhibited a wide range due to variations in sample types, prior treatments, and disease stages across the different studies.

## 2 DNA methylation in esophageal tissues

A total of 23 studies investigating the methylation patterns of EC using tissue samples were identified, encompassing a total of 62 genes. Among these studies, fresh frozen tissue (FFT) was the most frequently utilized sample type, followed by formalin-fixed and paraffin-embedded (FFPE) tissues. Additionally, a subset of studies employed endoscopic brushings to collect tissue samples. While two articles employed sequencing technology, the majority of studies employed MSP or qMSP as the primary research technique. A comprehensive summary of the results can be found in Table 1.

**TABLE 1:** The DNA methylation markers evaluated in esophageal tissues.

Markers	Year	Sample types	Sample size	DNA isolation method	DNA conversion method	Analytical method	Sensitivity (%)	Specificity (%)	AUC	Ref
*SFRP1, SFRP2, SFRP4, SFRP5*	2005	FFPE	40 EAC, 37 BE, 28 normal mucosa adjacent to BE, 30 SQ	QIAamp DNA Mini Kit	Self-made Reagent	MSP	SFRP1, 2, 4 and 5 were methylated in 92.5, 82.5, 72.5 and 85.0 of EAC; 81.1, 89.2, 78.4, 73.0 of BE; 25.0, 64.3, 32.1and 21.4 of normal mucosa adjacent to BE	90.0, 33.3, 100 and 86.7 for SFRP1, 2, 4 and 5	—	Zou et al. (2005)
*SFRP1*	2011	FFT	20 ESCC, 20 para-carcinoma tissue	TIANamp Genomic DNA Kit	CpGenome DNA Modification Kit	MSP	95.0	35.0	—	Meng et al. (2011)
*RASSF1A*	2005	FFT	55 ESCC	Self-made Reagent	Self-made Reagent	MSP	23.6	—	—	Yamaguchi et al. (2005)
*P16*	2022	FFPE, endoscopic brushings	1 ESCC, 12 LGD, 8 HGD, 30 esophagitis, 32 SQ	Com Win Biotech DNA extraction kit	EZ DNA Methylation-Gold Kit	qMSP	FFPE: LGD: 8.3, HGD: 12.5, ESCC: 30.4; endoscopic brushings: LGD: 25.0, HGD: 37.5, ESCC: 43.5	FFPE: 98.4, endoscopic brushings: 95.2	FFPE: 0.616 endoscopic brushings: 0.669	Fan et al. (2022)
*P16, DAPK, RAR-β, CDH1, RASSF1A*	2011	FFT	47 ESCC, 47 para-carcinoma tissue	QIAmp DNA Mini Kit	EZ DNA Methylation-Gold Kit	MSP	P16: 44.7, DAPK: 46.8, RAR-β: 46.8, CDH1: 42.6, RASSF1A: 14.9	P16: 78.7, DAPK: 87.2, RAR-β: 87.2, CDH1: 78.7, RASSF1A: 95.7	—	Li et al. (2011)
*P16, MGMT, hMLH1*	2008	FFT	125 ESCC, 125 para-carcinoma tissue, 10 SQ	—	Self-made Reagent	MSP	P16: 88.0, MGMT: 27.2, hMLH1: 3.2, three gene panel: 90.4	Para-carcinoma tissue: P16: 63.2, MGMT: 88.8, Hmlh1: 100.0, three gene panel: 56.8; SQ: 100.0 for individual gene and 3-marker panel	—	Wang et al. (2008)
*Reprimo*	2006	FFT	45 ESCC, 75 EAC, 25 BE, 11 HGD, 19 SQ	DNeasy Blood and Tissue Kit	Self-made Reagent	qMSP	BE: 36.0, HGD: 63.6, EAC:62.7, ESCC: 13.3	100.0	EAC: 0.812	Hamilton et al. (2006)
*TAC1*	2007	FFT	67 EAC, 24 ESCC, 60 BE, 40 dysplasias, 67 SQ	DNeasy Blood and Tissue Kit	Self-made Reagent	qMSP	BE: 63.3, dysplasias: 57.5, EAC: 61.2, ESCC: 50.0	92.5	EAC: 0.859, ESCC: 0.805	Jin et al. (2007)
*PTPRO*	2012	FFT	36 ESCC, 36 para-carcinoma tissue	ZR Genomic DNA II Kit	EZ DNA Methylation-Gold Kit	MSP	75.0	100.0	—	You et al. (2012)
*PKP1*	2012	—	56 EAC, 4 HGD, 39 BE, 55 SQ	InstaGene Matrix	—	MSP	33.9, 25.0 and 12.8 in EAC, HGD and BE	90.9	—	Kaz et al. (2012)
*RIZ1*	2012	FFT	47 ESCC, 47 para-carcinoma tissue, 47 SQ	DNeasy Blood and Tissue Kit	Self-made Reagent	MSP	55.3	Para-carcinoma tissue: 95.6, SQ: 100	—	Dong et al. (2012)
*TFPI2*	2012	FFPE	106 EC, 60 dysplasia, 9 SQ	Self-made Reagent	Self-made Reagent	MSP	Dysplasias: 30.0, EC: 67.0	100.0	—	Jia et al. (2012)
*ADHFE1, EOMES, SALL1, TFPI2*	2018	FFT	94 ESCC, 94 para-carcinoma tissue	Qiagen AllPrep DNA/RNA Mini Kit	EpiTect Fast DNA Bisulfite Kit	Targeted Bisulfite Sequencing	ADHFE1: 29.0, EOMES: 69.0, SALL1: 53.0, TFPI2: 50.0	ADHFE1: 94.0, EOMES: 77.0, SALL1: 90.0, TFPI2: 91.0	ADHFE1: 0.64, EOMES: 0.78, SALL1: 0.74, TFPI2: 0.71	Wang et al. (2018a)
*EPB41L3, GPX3, COL14A1*	2014	FFT	42 ESCC, 42 para-carcinoma tissue	QIAmp DNA Mini Kit	EZ-DNA Methylation-Gold Kit	MSP	EPB41L3: 59.5, GPX3: 54.8, COL14A1: 45.2	EPB41L3: 95.2, GPX3: 90.5, COL14A1: 88.1	—	Li et al. (2014)
*B3GAT2, ZNF793*	2015	Endoscopic brushings	10 BE and 44 SQ	DNeasy Blood and Tissue Kit	EZ DNA Methylation Kit	qMSP	B3GAT2: 50.0, ZNF793: 70.0	B3GAT2: 100.0, ZNF793: 100.0	B3GAT2: 0.946, ZNF793: 0.959	Yu et al. (2015)
*PAX1, ZNF582*	2017	FFPE	14 ESCC, 14 para-carcinoma tissue	iStat Nucleic Acid Extraction kit	iStat Bisulfite Conversion Kit	qMSP	PAX1: 100, ZNF582: 78.6	PAX1: 85.7, ZNF582: 100	PAX1: 0.893, ZNF582: 0.954	Huang et al. (2017)
*PAX1, SOX1, ZNF582*	2019	FFT	74 ESCC, 74 para-carcinoma tissue, 24 SQ	QIAamp DNA Mimi Kit	Qiagen®EpiTect Bisulfite Kit	Pyrosequencing	PAX1: 96.0, SOX1: 89.2, ZNF582: 93.2, 3-marker panel: 94.6	SQ; PAX1: 51.4, SOX1: 59.5, ZNF582: 75.7, 3-marker panel: 77.0	PAX1: 0.754, SOX1: 0.781, ZNF582: 0.898, 3-marker panel: 0.914	Tang et al. (2019)
*cg15830431, cg19396867, cg20655070, cg26671652, cg27062795*	2017	FFT	94 ESCC, 94 para-carcinoma tissue	Self-made Reagent	MethylMiner™ Methylated DNA Enrichment Kit	Targeted Bisulfite Sequencing	75.0	88.0	0.85	Pu et al. (2017)
*ARHGEF4, ELMO1, ST8SIA1, OPLAH, FER1L4, TBX15, ZNF671, IKZF1, TSPYL5, NDRG4, BMP3, DMRTA2*	2019	FFPE	41 EAC, 35 ESCC, 17 SQ	QIAamp FFPE Tissue Kit	EZ-96 DNA Methylation Kit	qMSP	—	—	EAC: ARHGEF4: 0.79, ELMO1: 0.99, ST8SIA1: 0.98, OPLAH: 0.94, FER1L4: 0.92, TBX15: 0.95, ZNF671: 0.89, IKZF1: 0.92, TSPYL5: 0.95, NDRG4: 0.96, BMP3: 0.96, DMRTA2:1.00 ESCC: ARHGEF4: 0.81, ELMO1: 0.74, ST8SIA1: 0.59, OPLAH: 0.77, FER1L4: 0.69, TBX15: 0.91, ZNF671: 0.89, IKZF1: 0.37, TSPYL5: 0.90, NDRG4: 0.54, BMP3: 0.50, DMRTA2:1.00	Qin et al. (2019)
*ZNF569*	2020	FFPE	86 ESCC, 56 SQ	FFPE RNA/DNA Purification Plus Kit	EZ DNA Methylation-Gold Kit	qMSP	69.3	90.0	0.847	Salta et al. (2020)
3-marker panel (*PAX9, SIM2, THSD4*)	2021	FFPE	132 ESCC and 36 SQ	Qiagen AllPrep DNA/RNA Mini Kit	EZ DNA Methylation-Gold Kit	Pyrosequencing	—	—	3-marker panel: 0.98	Talukdar et al. (2021)
12-marker panel (*MMP13, YEATS2, HDAC11, ZNF578, AFF3, PDE4D, SYNE3, SLC8A3, CPS1, HOXC10, LDB2, PACRG*)	2022	FFT	Training set: 60 ESCC, 60 para-carcinoma tissue Test set: 31 ESCC, 31 para-carcinoma tissue	Qiagen AllPrep DNA/RNA Mini Kit	—	450 K array	12-marker panel, Training set: 98.3 Test set: 96.8	12-marker panel, Training set: 93.3 Test set: 100.0	12-marker panel, Training set: 0.996 Test set: 0.971	Xi et al. (2022)
4-marker panel (*Up10, Up35-1, Cg6522, YPEL3*)	2022	Endoscopic brushings	Training set: 87 EAC, 19 BE, 20 LGD, 20 HGD, 48 SQ Test set: 40 EAC, 37 BE, 10 LGD, 15 HGD, 27 SQ	DNeasy Blood and Tissue Kit	EZ DNA Methylation Kit	Methylation-specific ddPCR	Training set: BE: 15.8, LGD: 50.0, HGD: 85.0, EAC: 90.8 Test cohort: BE: 32.4, LGD: 50.0, HGD: 80.0, EAC: 82.5	Training set: 97.9 Test set: 96.3	—	Yu et al. (2022)

EC, esophageal cancer; ESCC, esophageal squamous cell carcinoma; EAC, esophageal adenocarcinoma; FFT, fresh frozen tissue; FFPE, formalin-fixed and parrffin-embedded; HGD, high-grade dysplasia; LGD, low-grade dysplasia; SQ, normal squamous epithelium; BE, Barrett’s esophagus; MSP, methylation specific PCR; qMSP, quantitative methylation specific PCR; ddPCR, droplet digital PCR.

The selected literature spans from 2005 to 2022 and includes various studies on the performance of specific genes as methylation markers for early detection of EAC and ESCC. Zou et al. (2005) reported on the performance of methylated *SFRPs* (*SFRP1*, *SFRP2*, *SFRP4*, and *SFRP5*) in EAC detection, observing sensitivities of 92.5%, 82.5%, 72.5%, and 85.0% respectively. *SFRP1*, in particular, demonstrated high sensitivity (92.5%) and specificity (90.0%) as a potential single gene marker for EAC (Zou et al., 2005). Meng et al., 2011 conducted similar research on *SFRP1* for ESCC, yielding a sensitivity of 95.0% but a lower specificity of 35.0%. In contrast, Jin et al. (2007) reported on methylated *TAC1*, which exhibited a comforting specificity of 92.5% but a lower sensitivity of 61.2% for EAC screening (Jin et al., 2007). Li et al., 2011 highlighted *RASSF1A* as a marker for ESCC screening with a higher specificity of 95.7% but a relatively lower sensitivity of 14.9%. Huang et al., 2017 evaluated *PAX1* and *ZNF582* for ESCC detection, finding relatively balanced performances with sensitivities of 80.7% and 88.2% and specificities of 75.0% and 81.2% respectively. Tang et al. (2019) also investigated *PAX1* and *ZNF582*, reporting promising sensitivities of 96.0% and 93.2% and specificities of 51.4% and 75.7% respectively. Notably, when combined with *SOX1* as a panel, the sensitivity reached 94.6% and the specificity was 77.0% (Tang et al., 2019), suggesting the potential of combined methylation detection as a screening method. Subsequently, Xi et al., 2022 developed and validated a panel of 12 markers including methylated *MMP*, *YEATS2*, *ZNF578*, *AFF3*, and so on, demonstrating an impressive sensitivity of 96.8% and a specificity of 100%. The panel exhibited an area under the curve (AUC) of 0.971.

The detection of precursor lesions of EC has posed a persistent challenge over the years. In a study by Fan et al., a total of 52 samples of premalignant lesions, including LGD and HGD, were collected through endoscopic brushings to evaluate the detectability of methylated *P16*. The sensitivity for LGD and HGD was reported as 25.0% and 37.5% respectively, with a specificity of 95.2% (Fan et al., 2022). Yu et al. investigated a panel of methylated markers, namely, *Up10*, *Up35-1*, *Cg6522*, and *YPEL3*, using a similar methodology as Fan et al., aiming to screen for early-stage EC. They achieved higher sensitivities of 50.0% for LGD and 80.0% for HGD. However, the study did not provide specific information regarding the specificity of the panel (Yu et al., 2022).

Meanwhile, we have observed certain limitations in early studies focusing on the discovery of DNA methylation markers for EC using tissue samples. These issues include small sample sizes and significant imbalances between case and control groups, leading to potentially reduced research quality and result repeatability. For example, Yamaguchi et al., 2005 study solely comprised ECSS tissue samples, lacking any control subjects, thereby impeding an assessment of the specificity of *RASSF1A*. In another study by Jia et al., 2012, while including 106 EC samples, 60 dysplasia samples, and 9 SQ samples, the number of control samples was only about 1/12 of the EC samples, rendering it unsuitable for a valid case-control study. Furthermore, many of the identified methylation markers have not undergone replication or multicenter validation, which presents a challenge for subsequent translational studies based on such markers. Fortunately, in recent years, some studies have made progress in addressing these issues by including multiple-cohort validations (Xi et al., 2022; Yu et al., 2022).

## 3 DNA methylation in esophageal exfoliated cells

Esophageal balloon cytology was pioneered by Professor Qiong Shen, a renowned pathologist in China, during the 1960s. Initially employed for screening ESCC in Linxian, an area with a high incidence of the disease, this method yielded favorable outcomes (Yang and Chen, 2021). Some researchers have also explored the utilization of traditional esophageal balloons to collect esophageal exfoliated cells for methylation analysis, leading to the identification of several highly methylated genes, such as *P16*, within these cells (Roth et al., 2006; Adams et al., 2008). In recent years, more convenient and innovative devices for esophageal exfoliated cell collection have emerged, facilitating the early screening of esophageal cancer and its precancerous lesions, such as the Cytosponge (Paterson et al., 2020), EsophaCap (Zhou et al., 2019) and EsoCheck (Shahsavari et al., 2022).

In the review mentioned in Section 2, the tissue samples analyzed primarily consisted of patients with EC. However, research on methylation markers in esophageal exfoliated cells primarily focuses on the precancerous lesions, particularly in patients with BE (Table 2). Chettouh et al. investigated the methylation levels of four genes-*TFPI2*, *TWIST1*, *ZNF345*, and *ZNF569*-for BE screening in combination with Cytosponge. Cytosponge is a 30 mm compressed spherical sponge primarily designed with Trefoil factor 3 (TF-3) staining to aid in BE detection. (Fitzgerald et al., 2020). The individual sensitivities were 78.5%, 69.8%, 62.4%, and 59.1%, respectively, while the specificities ranged from 93.0% to 100% (Chettouh et al., 2018). The performance of *ZNF569* in detecting EC showed consistent results with Salta et al.'s study in tissue samples, with a sensitivity of 69.0% and a specificity of 90.0% (Salta et al., 2020). This suggests that *ZNF569* may serve as a promising methylation marker for EC screening, particularly when combined with other genes.

**TABLE 2:** The DNA methylation markers evaluated in esophageal exfoliated cells for EC early detection.

Markers	Year	Esophageal sampling device	Compliance rate (%)	Sample size	DNA isolation method	DNA conversion method		Analytical method	Sensitivity (%)	Specificity (%)	AUC	Ref
*P16, MGMT, RARß2, CLDN3, CRBP, MT1G*	2006	Esophageal balloon	—	12 ESCC	DNeasy Blood and Tissue Kit	Self-made Reagent		qMSP	P16: 16.7, MGMT: 33.3, RARß2: 16.7, CLDN3: 75.0, CRBP: 50.0, MT1G: 58.3	—	—	Roth et al. (2006)
4-marker panel (*AHRR, p16INK4a, MT1G, CLDN3*)	2008	Esophageal balloon	—	1 ESCC, 20 HGD, 26 MGD, 25 LGD, 25 esophagitis, 50 control	DNeasy Blood and Tissue Kit	EZ DNA-Methylation Gold kit		qMSP	50.0 for HGD	68.0	—	Adams et al. (2008)
*TFPI2, TWIST1, ZNF345, ZNF569*	2018	Cytosponge	—	149 BE, 129 control	QIAamp FFPE DNA Tissue Kit	EZ DNA-Methylation Gold kit		qMSP	TFPI2: 78.5, TWIST1: 69.8, ZNF345: 62.4, ZNF569: 59.1	TFPI2: 96.9, TWIST1: 93.0, ZNF345: 100, ZNF569: 99.2	TFPI2: 0.877, TWIST1: 0.814, ZNF345: 0.812, ZNF569: 0.787	Chettouh et al. (2018)
2-marker panel (*CCNA1, VIM*)	2018	EsoCheck	82.1	42 BE, 8 EAC and 36 control	DNeasy Blood and Tissue Kit	EpiTect Bisulfite Conversion Kit		Bisulfite sequencing–based methylation detection	88.1 for BE and 87.5 for EAC	91.7	CCNA1: 0.917, VIM: 0.908	Moinova et al. (2018)
2-marker panel *(CCNA1, VIM)*	2023	EsoCheck	96.3	Total of 275 subjects	DNeasy Blood and Tissue Kit	EpiTect Bisulfite Conversion Kit	Bisulfite sequencing–based methylation detection		—	—	—	Dan Lister et al. (2023)
4-marker panel (*P16, NELL1, AKAP12, TAC1*)	2019	EsophaCap	85.1	Training set: 13 BE with no dysplasia, 1 BE with LGD, 4 BE with HGD, 34 control Test set: 14 BE, 14 control	Methylation-on-beads method	Methylation-on-beads method		qMSP	Training set: 94.4 Test set: 78.6	Training set: 62.2 Test set: 92.8	Training set: 0.894 Test set:0.929	Wang et al. (2019)
2-marker panel *(VAV3, ZNF682)*	2018	EsophaCap	98.0	10 BE with no dysplasia, 5 BE with LGD, 4 HGD or EAC, 20 control	Puregene Buccal Cell Kit	EZ DNA Methylation Kit		qMSP	100 for all BE	100	1	Iyer et al. (2018)
5-marker panel (*VAV3, ZNF682, NDRG4, FER1L4, ZNF568*)	2020	EsophaCap	90.8	54 BE with no dysplasia, 20 BE with Indefinite dysplasia, 15 BE with LGD, 23 HGD or EAC, 89 control	Puregene Buccal Cell Kit	EZ DNA Methylation Kit		TELQAS	89.0 for BE without dysplasia, 95.0 for BE with any grade of dysplasia, 100.0 for EAC, 92.0 for all BE	94.0	0.97	Iyer et al. (2020)
5-marker panel (*VAV3, ZNF682, NDRG4, BMP3, ZNF568*)	2021	EsophaCap	—	Training set: 110 BE, 89 control Test set: 60 BE, 29 control	QIAsymphony DSP DNA Mini Kit	Hamilton STARlet liquid handling system		TELQAS	Training set: 93 Test set: 93	Training set: 90 Test set: 93	Training set: 0.96 Test set: 0.97	Iyer et al. (2021)
3-marker panel (*cg20655070, SLC35F1, and ZNF132*)	2022	EsophaCap	94.9	Training set: 22 ESCC, 44 control Test set: 13 ESCC, 15 control	DNeasy Blood and Tissue Kit	Methylation-on-beads method		qMSP	Training set: 86.0 Test set: 92.3	Training set: 86.0 Test set: 86.7	—	Ma et al. (2022)

EC, esophageal cancer; ESCC, esophageal squamous cell carcinoma; EAC, esophageal adenocarcinoma; HGD, high-grade dysplasia; MGD, mild-grade dysplasia; LGD, low-grade dysplasia; BE, Barrett’s esophagus; MSP, methylation specific PCR; qMSP, quantitative methylation specific PCR; TELQAS, target enrichment long-probe quantitative amplified signal.

EsophaCap is a 25 mm esophageal exfoliated cells collection device approved by the Food and Drug Administration (FDA) based on the 510 K guideline. Prasad et al. conducted an initial evaluation of the effect of sponge density (10 ppi vs. 20 ppi) and DNA yield on the analysis of DNA methylated markers (Iyer et al., 2018). They found that the10 ppi sponge resulted in minimal mucosal injury and yielded an abundant amount of DNA (approximately 38.0 μg per sample). Subsequently, they developed a 2-marker panel comprising *VAV3* and *ZNF682*, which exhibited a high AUC of 1.0 for detecting BE (Iyer et al., 2018). In 2020, Prasad et al. expanded the 2-marker panel to a 5-marker panel (*VAV3*, *ZNF682*, *NDRG4*, *FER1L4*, *ZNF568*) for the detection of BE and EAC, achieving sensitivities of 92.0% for BE and 100.0% for EAC (Iyer et al., 2020). A year later, they optimized this panel by replacing *FER1L4* with *BMP3* and validated it in two independent cohorts (Iyer et al., 2021). Furthermore, aside from BE, there is another feasibility study that utilizes EsophaCap for sample collection and early diagnosis of ESCC. This study developed a 3-marker panel (*cg20655070*, *SLC35F1*, and *ZNF132*) with sensitivities ranging from 86.0% to 92.0% and specificities ranging from 86.0% to 86.7% for ESCC (Ma et al., 2022).

EsoCheck, designed by PAVmed Inc., is an encapsulated, inflatable, and surface-featured balloon measuring 16 × 9 mm (Moinova et al., 2018). Differing from sponge-based collection devices, the balloon’s size is controlled through the injection or withdrawal of air using a syringe. Helen et al. developed a methylation panel combined with EsoCheck, named EsoGuard, which includes two markers (*CCNA1* and *VIM*). In a cohort of 86 individuals, EsoGuard achieved a sensitivity of 90.3% and specificity of 91.7% (Moinova et al., 2018). It is important to note that EsoGuard employs bisulfite Next-Generation Sequencing (NGS) as its detection method, while other DNA methylation detection methods for esophageal exfoliated cells are based on qMSP (Table 2). In 2023, a study of clinical utility of EsoGuard was proposed, and the results demonstrated of the overall concordance between EsoGuard results and upper endoscopy referral was 98.8% (Dan Lister et al., 2023).

Fortunately, studies related to DNA methylation markers in esophageal exfoliated cells are relatively more rigorous compared to those focusing on tissue samples, leading to more reliable results (Table 2). This robustness offers a solid foundation for the clinical application of this technology.

## 4 DNA methylation in blood

### 4.1 The blood DNA methylation markers for EC

Due to its convenience and non-invasiveness, the screening of blood markers is associated with better compliance compared to the other two methods. Our search yielded 11 articles encompassing 21 genes that utilized serum or plasma samples for EC early detection. The outcomes of these studies are summarized in Table 3.

**TABLE 3:** The blood DNA methylation markers for EC early detection.

Markers	Year	Sample types	Sample size	DNA isolation method	DNA conversion method	Analytical method	Sensitivity (%)	Specificity (%)	AUC	Ref
*P16*	2001	Serum*	31 ESCC, 40 control	Self-made Reagent	Self-made Reagent	MSP	22.6	100.0	—	Hibi et al. (2001)
*P16, E-cadherin, RAR*	2007	0.4 mL plasma	44 ESCC, 12 control	QIAmp DNA Blood Mini Kit	CpGenome DNA modification kit	MSP	P16: 13.6, E-cadherin: 9.1, RAR: 9.1, 3-marker panel: 31.8	100.0	—	Ikoma et al. (2007)
*RAR-β, DAPK, CDH1, P16, RASSF1A*	*2011*	Serum*	45 ESCC, 15 control	QIAmp DNA Blood Mini Kit	EZ-DNA Methylation-Gold Kit	qMSP	RAR-β: 26.7, DAPK: 73.3, CDH1: 84.4, p16: 6.7, RASSF1A: 62.2, 5-marker panel: 82.2	RAR-β: 86.7, DAPK: 86.7, CDH1: 80.0, p16: 100.0, RASSF1A: 93.3, 5-marker panel: 100.0	RAR-β: 0.567, DAPK: 0.800, CDH1: 0.822, p16: 0.533, RASSF1A: 0.778, 5-marker panel: 0.911	Li et al. (2011)
*TAC1*	2007	0.3 mL plasma	61 EAC, 20 dysplasia, 10 BE, 35 control	Self-made Reagent	Self-made Reagent	qMSP	EAC: 29.5, dysplasia: 0, BE: 0	91.4	—	Jin et al. (2007)
*PTPRO*	2012	Plasma*	36 ESCC, 10 control	ZR Genomic DNA II Kit	EZ DNA Methylation-Gold Kit	MSP	36.1	100.0	—	You et al. (2012)
*EPB41L3, GPX3, COL14A1*	2014	Plasma*	42 ESCC, 50 control	QIAmp DNA Blood Mini Kit	EZ-DNA Methylation-Gold Kit	MSP	EPB41L3: 31.0, GPX3: 40.5, COL14A1: 31.0, 3-marker panel: 64.3	100.0	EPB41L3: 0.655, GPX3: 0.702, COL14A1: 0.655, 3-marker panel: 0.821	Li et al. (2014)
*MGMT*	2014	Serum*	100 ESCC, 100 control	DNeasy Blood and Tissue Kit	Epitect Bisulphite Kit	MSP	70.0	—	—	Das et al. (2014)
*CASZ1, CDH13, ING2*	2018	0.2 mL plasma	10 ESCC, 3 control	QIAmp DNA Blood Mini Kit	EZ DNA Methylation kit	Matrix-assisted laser desorption/ionization time-of-flight mass spectrometry	All for 100	All for 100	All for 1	Wang et al. (2018b)
5-marker panel (*FER1L4, ZNF671, ST8SIA1, TBX15, ARHGEF4*)	2019	3–4 mL plasma	76 EAC, 9 ESCC, 98 control	Proprietary semiauto-mated silica bead DNA extraction method	Self-made Reagent	QuARTS assay	EC: 74.0 ESCC: 78.0	91.0	0.93 for all EC	Qin et al. (2019)
Targeted methylation panel	2021	Plasma*	Training set: 43 EC, 67 controlTest set: 42 EC, 68 control Validation set: 83 EC, 98 control	QIAamp Circulating Nucleic Acid Kit	EZ-96 DNA Methylation-Lightning™ MagPrep	Deep targeted bisulfite sequencing	Training set: 86.0 Test set: 76.2 Validation set: 74.7	Training set: 94.0 Test set: 94.1 Validation set: 95.9	Training set: 0.963 Test set: 0.932 Validation set: 0.943	Qiao et al. (2021)
*SEPT9*	2022	3.5 mL plasma	188 EC, 125 benign esophageal diseases, 270 control	BioChain plasma processing kit	BioChain Bisulfite Conversion Kit	qMSP	43.1	92.6	0.69	Zhang et al. (2022)
2-marker panel *(KCNA3 and OTOP2)*	2023	Plasma*	Training set: 53 ESCC, 176 control Validation set: 65 ESCC, 155 control	Wuhan Ammunition Life Science and Technology Co., Ltd., Nucleic Acid Extraction and Purification Kit	Wuhan Ammunition Life Science and Technology Co., Ltd., Bisulfite Conversion Kit	qMSP	Training set: 84.9 Validation set: 81.5	Training set: 94.3 Validation set: 92.9	Training set: 0.91 Validation set: 0.88	Bian et al. (2023)
2-marker panel *(ZNF582 and FAM19A4)*	2023	0.5–1 mL plasma	Training set: 48 EC, 101 control Validation set: 20 EC, 20 control	Versa-Auto-pure nucleic acid purification system	VersaBio fast bisulfite conversion kit	qMSP	Training set: 60.4 Validation set: 60.0	Training set: 83.2 Validation set: 90.0	Training set: 0.673 Validation set: 0.845	Pei et al. (2023)

EC, esophageal cancer; ESCC, esophageal squamous cell carcinoma; EAC, esophageal adenocarcinoma; BE, Barrett’s esophagus; MSP, methylation specific PCR; qMSP, quantitative methylation specific PCR; QuARTS, quantitative allele-specific real-time target and signal amplification; BSP, bisulfite sequencing PCR. * Without the description for the volume of serum or plasma.

Daito et al. conducted a study in 2001 using serum methylated *P16* to detect early EC, but achieved a low sensitivity of 22.6% (Hibi et al., 2001). In another study by Jin et al., 2007, the evaluation of *TAC1* methylation for EAC was performed on both plasma and tissue samples simultaneously. The results indicated that *TAC1* exhibited similar specificity in plasma and tissue (91.4% vs. 92.5%), but the sensitivity significantly decreased (29.5% vs. 61.2%). Similar findings were observed in Yan et al.'s study, where the sensitivity of methylated *PTPRO* was 75.0% in tissue but only 36.1% in plasma (You et al., 2012). The lower sensitivity of methylation markers in blood compared to tissue is attributed to the lower abundance of circulating tumor DNA (ctDNA) in the blood. Nevertheless, there are still individual gene markers, such as *MGMT* reported by Das et al., 2014, which demonstrate good sensitivity of 70.0%. However, employing a marker panel appears to be a preferable approach for blood-based methylation screening. Li et al., 2011 evaluated the detection efficiency of 5 methylated genes, including *P16*, *DAPK*, *RAR-β*, *CDH1*, and *RASSF1A*, in serum in 2011. When considering single gene detection, the sensitivity of these 5 genes was 6.7%, 73.3%, 26.7%, 84.4%, and 62.2%, respectively, with corresponding specificities of 100%, 86.7%, 86.7%, 80.0%, and 93.9%. However, when these 5 genes were combined, the sensitivity increased to 82.8% with a specificity of 100%. Qin et al. identified 23 candidate methylation markers from tissue samples that exhibited sensitivity for both EAC and ESCC. Subsequently, they selected 12 methylation markers for plasma testing and narrowed down to 5 markers (*FER1L4, ZNF671, ST8SIA1, TBX15, ARHGEF4*) to develop a panel for detecting both EAC and ESCC. This panel demonstrated sensitivities of 74% for EAC and 78% for ESCC, with a specificity of 91% (Qin et al., 2019). However, the sensitivity of the 5-gene panel in detecting stage I EC was only 43% (Qin et al., 2019). Bian et al. selected 2 markers (*KCNA3* and *OTOP2*) from 5 methylation markers and validated them in both the training and validation sets. The 2-marker panel demonstrated good diagnostic performance for ESCC in both the training and validation sets, with AUCs of 0.91 and 0.88, respectively. Additionally, it showed a sensitivity of 78.4% for stage I-II ESCC (Bian et al., 2023). Pei et al. developed a 2-marker panel using *ZNF582* and *FAM19A4* in 0.5–1 mL of plasma, but the overall sensitivity and specificity were not high (Table 3) (Pei et al., 2023).

In addition to the commonly used MSP and qMSP, mass spectrometry and NGS have also been utilized in the detection of EC in blood samples. For instance, Wang et al. constructed a 3-gene panel (*CASZ1, CDH13, ING2*) using matrix-assisted laser desorption/ionization time-of-flight mass spectrometry and validated it with 0.2 mL plasma samples, achieving an overall AUC of 1.0 (Wang et al., 2018). However, this study only included 10 cases of ESCC and 3 control cases, necessitating more samples to further evaluate the technology (Wang et al., 2018). On the other hand, Qiao et al. identified 921 differentially methylated regions based on tissue samples and constructed a plasma diagnostic model for EC by using deep targeted bisulfite sequencing. They tested the model in three independent cohorts and achieved good sensitivities (74.7%–86.0%) and specificities (94.0%–95.9%) (Qiao et al., 2021). However, the sensitivity of this diagnostic model for stage 0-II esophageal cancer was only 58.8% (Qiao et al., 2021).

Additionally, it is worth mentioning that *SEPT9*, a widely used detection marker for colorectal cancer (CRC) (Zhao et al., 2019; Zhang et al., 2021), exhibited promising specificity of 92.6% for EC detection (Zhang et al., 2022).

### 4.2 The blood DNA methylation markers for pan-cancer

The detection of multiple cancer types through the use of individual methylated genes or a panel of methylated genes, referred to as a pan-cancer test, represents a novel approach aimed at reducing cancer morbidity and mortality (Duffy et al., 2021). This review provides a summary of seven pan-cancer tests utilizing methylation markers, which have been applied to at least two cancer types, including EC (Table 4).

**TABLE 4:** The pan-cancer DNA methylation markers for EC early detection.

Markers	Year	Sample types	Cancer types	Sample size	DNA isolation method	DNA conversion method	Analytical method	Sensitivity for EC (%)	Specificity (%)	AUC for EC	Ref
*SFRP1*	2015	Serum*	ESCC, GC	36 ESCC, 42 GC, 42 control	Axygen blood mini kit	Sigma DNA methylation kit	MSP	31.0	88.1	—	Liu et al. (2015)
A panel consisting of 11,787 CpG sites	2020	1 mL plasma	EC, GC, HCC, LC, CRC	113 EC, 104 GC, 52 HCC, 103 LC, 42 CRC, 414 control	QIAamp Circulating Nucleic Acid kit	Methylcode Bisulfite Conversion Kit	Targeted bisulfite sequencing	91.0	94.7–96.1	—	Chen et al. (2020)
SEPT9	2020	3.5 mL plasma	EC, GC, HCC, CRC	106 EC, 239 GC, 128 HCC, 291 CRC, 423 precancerous diseases, 843 control	BioChain plasma processing kit	BioChain Bisulfite Conversion Kit	qMSP	42.6	94.6	0.69	Song et al. (2020)
A panel consisting of 1,116,720 CpG sites	2020	10 mL plasma	12 cancer types (anus, bladder, colon/rectum, esophagus, head and neck, liver/bile-duct, lung, lymphoma, ovary, pancreas, plasma cell neoplasm, stomach)	Trianing set: 1531 cancer (including 50 EC), 1521 non-cancer, Validation set: 654 cancer (including 21 EC), 610 non-cancer	QIAamp Circulating Nucleic Acid kit or a modified Automated MagMax kit	EZ-96 DNA Methylation Kit	Targeted bisulfite sequencing	Trianing set: 82.0 Validation set: 81.0	Trianing set: 99.8 Validation set: 99.3	—	Liu et al. (2020b)
A panel of >100,000 methylation regions	2021	Plasma*	12 cancer types (anus, bladder, colon/rectum, esophagus, head and neck, liver/bile duct, lung, lymphoma, ovary, pancreas, plasma cell neoplasm, and stomach)	2823 cancer (including 85 EC), 1254 control	Automated MagMax kit	—	Targeted bisulfite sequencing	85.0	99.5	—	Klein et al. (2021)
A panel consisting of 10,677 differentially methylated regions	2021	1–2 mL plasma	EAC, ESCC, PDAC, HCC, CRC, GC	12 EAC, 48 ESCC, 74 PDAC, 43 HCC, 40 CRC, 37 GC, 46 control	QIAamp Circulating Nucleic Acid kit	EZ DNA-Methylation Gold kit	Targeted bisulfite sequencing	—	—	ESCC: 0.94, EAC: 0.90	Kandimalla et al. (2021)
3-marker panel (*ZNF582, ELMO1, TFPI2*)	2022	3.5 mL plasma	EC, EJC, GC	48 EC, 29 EJC, 109 GC, 190 control	Versa-Auto-pure nucleic acid purification system	VersaBio fast bisulfite conversion kit	qMSP	79.2	90.0	0.893	Peng et al. (2022)
6-marker panel *(KCNQ5, C9orf50, CLIP4, ELMO1, ZNF582 and TFPI2)*	2023	3.5 mL plasma	EC, GC, CRC	Training set: 17 EC, 39 GC, 40 CRC, 51 control Validation set: 18 EC, 40 GC, 24 CRC, 75 control	Versa-Auto-pure nucleic acid purification system	VersaBio fast bisulfite conversion kit	qMSP	Training set: 64.7 Validation set: 83.3	Training set: 94.1 Validation set: 86.7	Training set: 0.937 Validation set: 0.921	Dai et al. (2023)
A panel of 161,984 CpG sites	2023	Plasma	EC, HCC, LC, CRC, PDAC, OC	Training set: 50 EC, 76 HCC, 65 LC, 87 CRC, 64 PDAC, 57 OC, 626 control Validation set: 64 EC, 66 HCC, 42 LC, 32 CRC, 53 PDAC, 44 OC, 123 control Independent validation set: 47 EC, 82 HCC, 121 LC, 59 CRC, 91 PDAC, 73 OC, 473 control	QIAamp Circulating Nucleic Acid Kit	—	Targeted bisulfite sequencing	Training set: 80.0 Validation set:73.4 Independent validation set: 59.5	Training set: 99.7 Validation set: 100.0 Independent validation set: 98.9	—	Gao et al. (2023)

AUC, area under the curve; GC, gastric cancer; CRC, colorectal cancer; EC, esophageal cancer; HCC, hepatocellular carcinoma; LC, lung cancer; PDAC, pancreatic adenocarcinoma; EJC, esophagogastric junction cancer; ESCC, esophageal squamous cell carcinoma; EAC, esophageal adenocarcinoma; OC, ovary cancer; MSP, methylation specific PCR; qMSP, quantitative methylation specific PCR. * Without the description for the volume of serum or plasma.

In the detection of ESCC and GC, Liu et al., 2015 reported a sensitivity of 31.0% for EC and a specificity of 88.1% using methylated *SFRP1* in serum. Similarly, Song et al., 2020 used methylated *SEPT9* in plasma for the detection of four cancers (EC, gastric cancer [GC], hepatocellular carcinoma [HCC], and CRC) and achieved a higher sensitivity and specificity of 42.6% for EC and 94.6% respectively. Peng et al., 2022 developed a panel combining *ZNF582*, *ELMO1*, and *TFPI2*, which allowed for the simultaneous detection of GC, EC, and esophagogastric junction cancer (EJC), with a sensitivity of 79.2% for EC and a specificity of 90.0%. In 2023, Dai et al., 2023 developed a 6-marker panel (*KCNQ5*, *C9orf50*, *CLIP4*, *ELMO1*, *ZNF582* and *TFPI2*) to detect of EC, GC and CRC, it achieved sensitivities for detecting EC of 64.7% and 83.35 in training and validation sets with specificities of 94.1% and 86.7%.

In 2020, GRAIL, Inc. published a novel multi-cancer detection panel consisting of 1,116,720 CpG sites by using the cfDNA in 10 mL plasma, for detecting 12 types of cancer, including EC, head and neck cancer, CRC, and lung cancer, they validated this panel in two large cohorts and achieved 82.0% and 81.0% sensitivities in training and validation sets, with specificities of 99.8% and 99.3%, respectively, while the sensitivity for stage I EC was only 16.7% (Liu et al., 2020). Next year, Klein et al. optimized this panel and also detecting 12 types of cancer by using a panel of over 100,000 methylation regions in plasma. They obtained a sensitivity of 85.0% for EC and a specificity of 99.5%, but the sensitivity for stage I EC was as less as 12.5% (Klein et al., 2021). It is worth noting that, despite the introduction of the cancer signal origin function in this panel, it is still unable to distinguish between EC and GC (Liu et al., 2020; Klein et al., 2021). Kandimalla et al. developed a targeted bisulfite sequencing-based panel for detecting five types of cancer, utilizing a reduced volume of plasma (Kandimalla et al., 2021). They achieved impressive AUC values of 0.94 for ESCC and 0.90 for EAC. However, it is worth noting that they did not assess the performance of the panel in detecting early-stage ESCC and EAC (Kandimalla et al., 2021). Furthermore, this panel demonstrated high accuracy in distinguishing between ESCC/EAC and other digestive tract cancers (Kandimalla et al., 2021). In 2023, the data of a large clinical trial (called The THUNDER study) for a customized panel with 161,984 CpG site for detecting six types of cancers was published, it can detect 59.5%–80.0% EC in three cohorts with super high specificities, but the sensitivities stage I EC still relatively low (Gao et al., 2023).

## 5 DNA isolation and conversion methods for DNA methylation analysis

Currently, the most commonly used method for DNA methylation analysis is still based on bisulfite conversion. Therefore, DNA extraction and conversion are the two major pre-analytical steps that have the greatest impact on DNA methylation detection. In this review, the DNeasy Blood and Tissue Kit is mentioned as the most commonly used kit for DNA isolation from tissue or esophageal exfoliated cells samples (Table 1 and 2). On the other hand, for the isolation of cfDNA from blood, the QIAamp Circulating Nucleic Acid Kit is the most frequently used kit (Table 3, 4). As for the DNA conversion process, regardless of the sample type, there is a preference for using the EZ DNA Methylation-Gold Kit (Table 1–4). The volume of plasma or serum is another crucial factor affecting cfDNA isolation and DNA methylation analysis. However, upon reviewing the literature summarized in Table 3, 4, it became apparent that many studies lacked sufficient details about the plasma volume used in their methods description. As a consequence, subsequent researchers might face challenges when attempting to replicate these studies.

## 6 Discussion

### 6.1 The effect of sample types on DNA methylation for detection of esophageal cancer

EC is a highly lethal disease associated with a poor prognosis, emphasizing the importance of early screening to improve patient survival rates and quality of life. DNA methylation, a widely studied epigenetic modification, is considered a promising tool for cancer screening due to its common occurrence, early onset, and stability during tumorigenesis (Ruddon, 2010). It can be detected in various sample types, including tissues, exfoliated cells, and body fluids such as blood, stool, urine, and cerebrospinal fluid (Li et al., 2014; De Mattos-Arruda et al., 2015; Wang et al., 2015; Husain et al., 2017; Moinova et al., 2018). In this review, we summarized the literature pertaining to DNA methylation detection in EC with different sample types, and assessed the potential and challenges of using DNA methylation as an early detection/screening tool for EC. For those sample types, DNA methylation in tissue is no a suitable sample for EC early detection, because it is an invasive sample, which mostly be used for pathological diagnosis and the discovery stage of DNA methylation markers (Table 5). While esophageal exfoliated cells and blood are two recommended sample types for EC early detection, although the sensitivity and specificity of DNA methylation marker in blood are lower than those in esophageal exfoliated cells, but the high compliance of blood will increase participation rate in early screening of EC (Table 5).

**TABLE 5:** The summary of DNA methylation in different sample types for EC early detection.

Sample types	Is it suitable for early detection	Sensitivity	Specificity	Compliance
Tissue	No	High	High	Low
Esophageal exfoliated cells	Yes	High	High	Medium
Blood	Yes	Medium	Medium	High

Theoretically, the methylation level in cancer tissue samples should be higher compared to other body fluid samples, such as blood, as suggested by previous studies. This is because the ctDNA in the blood originates from apoptotic cancer tissue, and this only accounts for a small portion of the cancer tissue. After entering the bloodstream, ctDNA undergoes systemic dilution, resulting in lower concentrations in blood. In the extracted blood cfDNA, only a small fraction is ctDNA, with the majority derived from normal cells (Thierry et al., 2016). Therefore, most of the reviewed literature in this study supports this observation. However, there may be exceptions in individual studies. Li et al. evaluated the performance of detecting EC using five methylated genes (*P16*, *DAPK*, *RAR-β*, *CDH1*, *RASSF1A*) in both tissue and blood samples. They found that only *P16* and *RAR-β* exhibited higher sensitivity in tissues compared to blood, while the other three genes showed the opposite trend (Li et al., 2011).

The accuracy of the DNA methylation test can be influenced by the selection of target CpG sites and the design of the panel. It is crucial to ensure that the chosen CpG sites are informative and specific for the target cancer types, and that the panel design is optimized for sensitivity and specificity. In addition, significant differences were observed when comparing the detection ability of the same gene in tissue across two different studies. As shown in Table 1, in Zou et al., 2005 study, the sensitivity and specificity of *SFRP1* were reported as 92.5% and 90%, respectively. However, in the study conducted by Meng et al., 2011 the sensitivity and specificity of *SFRP1* were 95.0% and 35.0%. It is worth noting that the experimental group in Zou et al.'s study consisted of patients with EAC and BE, whereas in Meng et al.'s study, the experimental group comprised patients with ESCC. EAC and ESCC are two completely different types of cancer in terms of molecular subtyping. The clinical treatment strategies for these two cancers are also completely different (Cancer Genome Atlas Research NetworkAnalysis Working Group: Asan UniversityBC Cancer AgencyBrigham and Women’s HospitalBroad InstituteBrown University et al., 2017). Therefore, it is not surprising that the observed significant differences in *SFRP1* methylation in EAC and ESCC were found in the aforementioned studies. When comparing the performance of the same marker, it is essential to consider whether the enrolled subjects are consistent. Different subtypes of EC represent distinct origins, resulting in varying sensitivities even when examining the same gene and methylation site. Additionally, the selection of the control group is of great importance. Zou et al.'s study employed normal squamous (SQ) esophageal tissue as the control group, while Meng et al. used para-carcinoma tissue. This selection significantly impacts the specificity of detection. It is observed that many studies did not clearly define the scope of para-carcinoma tissue, making it challenging to establish a uniform comparison across articles and differentiate between para-carcinoma tissue and SQ. Consequently, the lack of consistency in control group selection directly affects the longitudinal comparison of results.

**FIGURE 1:**
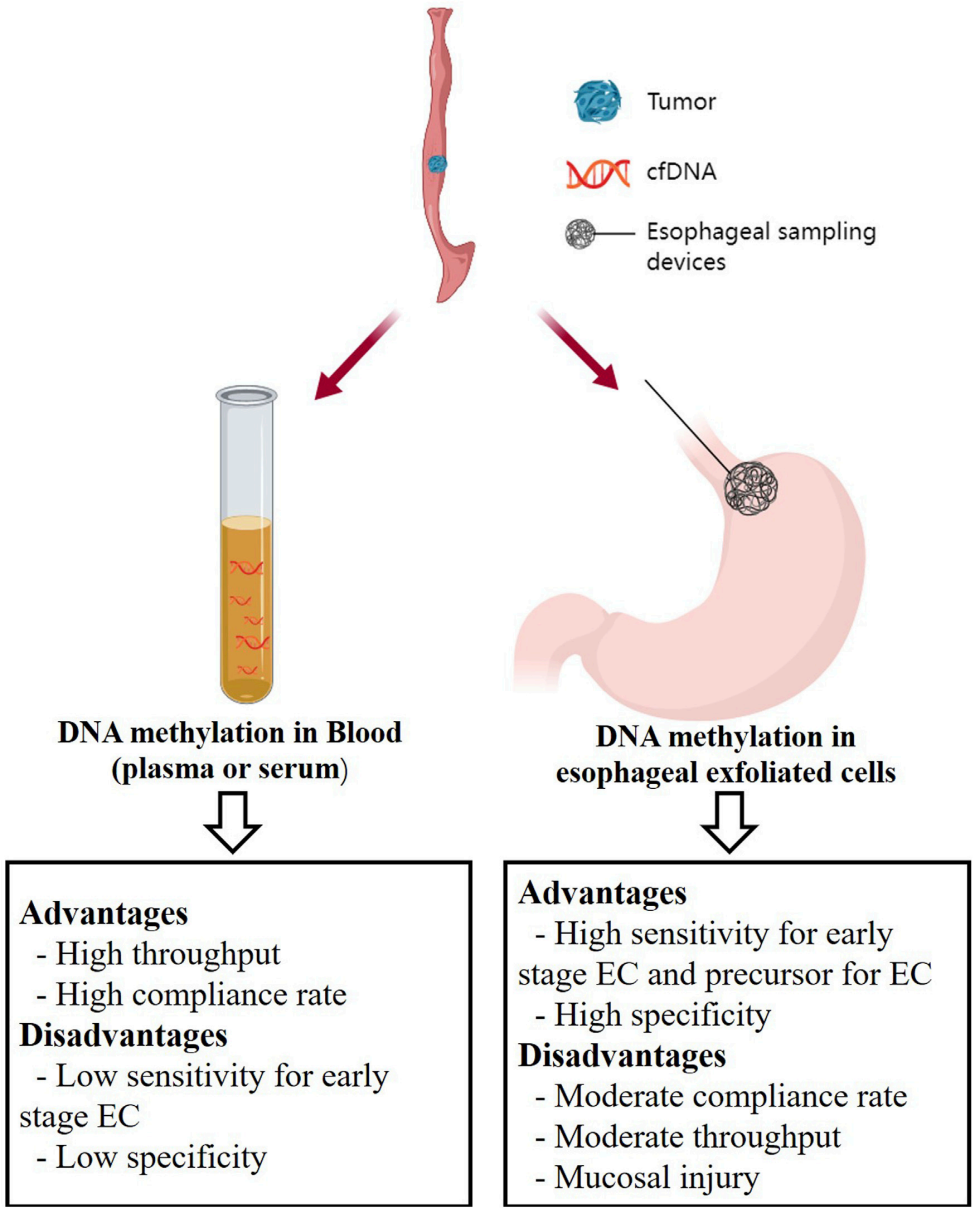
The non-endoscopic methods for EC early detection. Created with MedPeer (www.medpeer.cn).

Esophageal balloon is an emerging diagnostic device for EC. This method utilizes capsule sponge-on-string devices specifically designed to capture cells from the esophageal mucosa, offering a direct sampling of the affected tissue. Patients swallow these esophageal sampling devices, which consist of a sponge attached to a string. After a few minutes, the device is retrieved through the mouth, and the sponge is then examined for the presence of cancer cells or DNA (Iyer et al., 2020). Compared to traditional endoscopy, this technique inherits the advantages of providing a direct sample of the affected tissue, thereby potentially increasing sensitivity and specificity while minimizing invasiveness and improving patient compliance. Moreover, esophageal sampling devices can collect millions of cells (Wang et al., 2019), resulting in higher sensitivity compared to cfDNA-based method, especially in detecting early-stage EC. Meanwhile, since the samples collected by esophageal sampling devices are exclusively from the esophagus, they avoid interference from other organs during DNA methylation analysis, resulting in high specificity (Figure 1). For instance, DNA methylation markers such as *TFPI2*, *NDRG4*, and *BMP3* have been identified as effective markers for early detection/screening of CRC in blood or stool (Imperiale et al., 2014; Rasmussen et al., 2016; Rokni et al., 2018). However, when detecting these markers in esophageal exfoliated cells collected using esophageal sampling devices (Table 2), we can confidently attribute these methylation signals to the esophagus rather than the colon. Furthermore, it is important to note that esophageal sampling devices are more invasive compared to cfDNA-based methods, necessitating specialized equipment and trained personnel for the procedure. Additionally, the risk of complications such as bleeding, mucosal injury or perforation cannot be ignored (Figure 1) (Iyer et al., 2018; Januszewicz et al., 2019). Therefore, the utilization of esophageal sampling devices still has an approximate noncompliance rate of 10% during the application (Table 2). However, current DNA methylation studies based on the esophageal exfoliated cells mainly focus on EAC and its precursor lesions, with only one study specifically targeting ESCC, and a lack of validation for ESCC precursor lesions (Ma et al., 2022). ESCC constitutes the majority of EC cases (Yang et al., 2020), making it essential to pay more attention to the methylation analysis using esophageal exfoliated cells in ESCC in future research. This would provide a feasible pathway for early prevention of ESCC.

Blood testing, as a non-invasive method, offers high compliance. Compared to other approaches, the collection and processing of blood is a routine procedure, and DNA methylation analysis can be carried out using standard laboratory techniques with high-throughput, which may be more cost-effective than obtaining esophageal exfoliated cells (Figure 1) (Wang et al., 2021). Blood testing not only serves as a screening tool for cancer but also allows for more frequent and safer monitoring of response to anticancer therapies in clinical practice (Rothwell et al., 2019). However, cfDNA-based methods have a notable drawback, lacking specificity due to the interference from other organs. They can detect DNA fragments released from non-cancerous cells or other cancer tissues, leading to false positives. Furthermore, the sensitivity of DNA methylation markers in blood, particularly in early-stage disease, regardless of the detection approach, is relatively low (Table 3, 4). This limitation hinders their clinical utility (Figure 1).

Plasma is more commonly utilized than serum, likely due to the lower fraction of ctDNA in serum compared to plasma, as well as higher background noise and larger DNA fragments, as supported by numerous previous studies (Lee et al., 2020; Pittella-Silva et al., 2020). Moreover, there is considerable variation in plasma volumes reported in the literature, ranging from 0.2 to 3.5 mL, with some studies lacking sufficient explanation (Table 3, 4). This disparity in plasma volume can significantly impact sensitivity comparisons. Insufficient plasma volume may result in lower cfDNA yield and poorer quality, ultimately compromising the sensitivity and specificity of methylation analysis. Therefore, in clinical applications, it is often necessary to draw a larger amount of blood to improve sensitivity. However, excessive blood collection may cause discomfort or reluctance among participants. Thus, current cfDNA methylation testing typically recommends drawing 10 mL of blood, from which 3–4 mL of plasma is separated for subsequent analysis.

Throughout the literature reviewed in this review, *ZNF582* and *TFPI2* emerged as highly promising DNA methylation markers for early detection of EC. *ZNF582* was reported twice in tissue samples (Table 2), with both studies indicating good sensitivity and specificity (Huang et al., 2017; Li et al., 2019). Additionally, a study using plasma samples showed *ZNF582*s favorable sensitivity in detecting EC (Peng et al., 2022; Bian et al., 2023; Dai et al., 2023; Pei et al., 2023). Similarly, *TFPI2* has been validated in tissue samples (Jia et al., 2012), esophageal exfoliated cells (Chettouh et al., 2018), and plasma samples (Peng et al., 2022; Dai et al., 2023) from EC patients. However, both *ZNF582* and *TFPI2* face a common challenge: they are not specific to EC as methylation markers. For instance, *ZNF582* exhibits high methylation levels in GC and cervical cancers (Li et al., 2019; Peng et al., 2022), while *TFPI2* displays elevated methylation in GC and CRC (Hibi et al., 2011; Peng et al., 2022). When detecting these markers in esophageal epithelial exfoliated cells, interference from other organs is minimized. Yet, when using blood as the testing sample, interference from other organs may result in false positives. In fact, most blood DNA methylation markers are considered pan-cancer markers. For instance, *SEPT9* shows methylation positivity in plasma samples from CRC, GC, EC, HCC, and cervical cancers (Potter et al., 2014; Oussalah et al., 2018; Cao et al., 2020; Zhang et al., 2022; Bu et al., 2023), while *P16* also serves as a common pan-cancer methylation marker (Hibi et al., 2001; Zou et al., 2002; Hou et al., 2005; Lou-Qian et al., 2013). Therefore, detecting DNA methylation markers in EC blood samples often requires specific population screening or the use of tissue-origin algorithms to avoid false positive signals. Meanwhile, in blood sample testing, the future trend will likely involve the combination of multiple DNA methylation markers to enhance sensitivity for early-stage cancer detection.

### 6.2 The effect of analytical methods on DNA methylation for detection of esophageal cancer

In fact, apart from sample volume, there are several other sample preprocessing steps that significantly impact the performance of blood DNA methylation tests, such as the use of a preservation solution before freezing or centrifugal treatment after plasma collection can play a role (Kerachian et al., 2021). Additionally, the storage temperature of samples (ranging from −80°C to 4°C) and the time interval between sample collection and cryopreservation (ranging from 30 min to 24 h or even until the sample is tested) can also influence the results (Kerachian et al., 2021). The extraction and conversion methods of DNA, as described in the literature (whether using self-made reagents or commercialized kits), can affect the amount of DNA extracted, thereby potentially impacting the detection sensitivity and specificity. Studies have demonstrated notable differences in cfDNA recovery efficiency and bisulfite conversion efficiency among various cfDNA isolation kits and bisulfite conversion kits (Sorber et al., 2017; Worm Ørntoft et al., 2017). Therefore, standardizing the operating procedures and implementing quality control measures are crucial to ensure accurate and reliable test results.

MSP, qMSP, and bisulfite NGS are three commonly used methods for methylation analysis. MSP, a traditional method, has been widely employed in various sample types due to its ease of use and low cost. However, its sensitivity and specificity are limited by the potential for cross-contamination and the inability to detect low-frequency methylated DNA (Mao and Chou, 2010; Ramalho-Carvalho et al., 2018). Moreover, MSP lacks the capability to quantitatively assess markers, which is a significant drawback. On the other hand, qMSP, a modified method combining MSP and qPCR, allows for quantitative analysis while minimizing cross-contamination. Nevertheless, it is also limited to a small number of CpG sites and has difficulty detecting low-frequency methylated DNA (Sigalotti et al., 2019). Currently, several FDA and Chinese National Medical Products Administration (NMPA) approved non-invasive cancer early detection tests are based on qMSP, which are valued for their cost-effectiveness and convenience (Potter et al., 2014; Wu et al., 2016; Wang et al., 2020). Notably, the commercially available Epi proColon kit, which examines *SEPT9* methylation in blood, has achieved significant success in cancer detection (Potter et al., 2014; Song and Li, 2015). In contrast, bisulfite NGS, a high-throughput analytical method for DNA methylation markers, has not demonstrated a significant advantage over qMSP-based approaches. Its high cost and complex operational process have limited its widespread application (Luo et al., 2020).

Single cancer tests are specifically designed to detect a particular type of cancer, such as CRC in the case of the Epi Procolon test. However, they are not intended to detect other types of cancer, which can be a limitation when a patient has a different cancer type or multiple cancers. Additionally, the cost of single cancer tests can be prohibitive, posing a barrier to access for patients who cannot afford them or for healthcare systems with limited resources. In contrast, pan-cancer tests have the ability to detect multiple cancers in a single tube reaction, offering the potential to revolutionize cancer detection and treatment. This review summarizes several studies on pan-cancer detection, and Table 4 demonstrates their noteworthy performance. This approach presents a novel concept for future cancer screening in specific systems, such as gastrointestinal cancers or gynecologic cancers. However, how to enhance the sensitivity of pan-cancer tests for early-stage cancer diagnosis and effectively reduce the testing cost remains a challenge that needs to be addressed in future research.

### 6.3 Future and limitation

**FIGURE 2:**
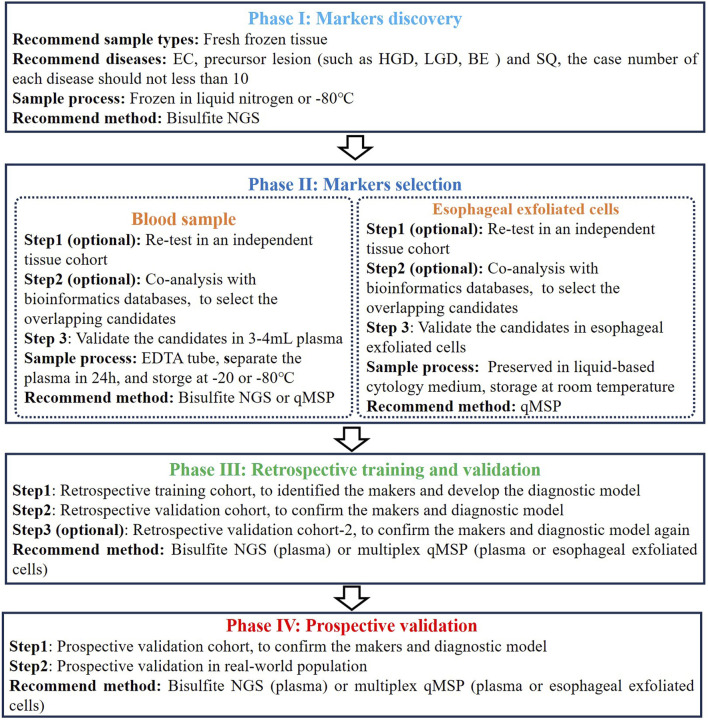
The flowchart for developing and validating a DNA methylation assay for early EC detection.

Based on the different sample types and analytical methods mentioned above, we summarized a flowchart suitable for developing and validating a DNA methylation assay for early EC detection (Figure 2). In the initial phase of marker discovery (Phase I), it is advisable to utilize FFT samples instead of FFPE samples to mitigate potential DNA degradation and loss, thus minimizing information loss. This stage necessitates the inclusion of diverse sample and disease types, encompassing a comprehensive range of EC samples across various stages, while ensuring age consistency between the EC and control groups, to identify the most specific candidates. NGS stands out as the optimal method for marker discovery due to its high throughput and potential for novel markers.

During the subsequent phase of marker selection (Phase II), it is advisable to validate the initially selected overlapping candidates across additional tissue cohort or databases to mitigate candidate preference. Subsequently, candidate validation should be performed using blood or esophageal exfoliated cells, as methylation levels in tissues may not entirely correlate with those in plasma or esophageal exfoliated cells, especially in plasma samples. Consistent with tissue validation, age, disease type, cancer stage, and cancer location distributions in plasma or esophageal exfoliated cell cohorts should be uniform. Concurrently, maintaining consistency in sample processing methods throughout assay development is essential. Despite the abundance of commercial DNA extraction and methylation conversion kits, thorough validation across multiple batches is imperative to ensure result robustness. Regarding plasma samples, sampling is typically conducted using EDTA tubes, which should be stored at room temperature for a maximum of 4 h or at 2°C–8°C for no more than 24 h, with plasma separation completed within the latter timeframe. Currently, cfDNA collection tubes permit blood samples to be stored at room temperature for up to 7 days before plasma separation, thereby enhancing the convenience of plasma-based detection methods (Hidestrand et al., 2012). Esophageal exfoliated cells are typically preserved in liquid-based cytology medium, such as PreservCyt medium (Hologic, Inc., Marlborough, MA, United States), and can be stored at room temperature for up to 1 month.

Subsequently, the remaining candidates are utilized in constructing the diagnostic model (Phase III). This step necessitates incorporating an adequate number of early EC and control samples, along with precancerous lesion and interfering samples, to derive relatively accurate diagnostic models and cut-off values. While combining more markers often yields higher sensitivity, in light of comprehensive costs and routine screening practices’ accessibility, we advocate employing multiplex qMSP methods for model construction. Following the establishment of the diagnostic model, it is imperative to validate its accuracy and repeatability once more across an adequate number of validation cohorts. It is worth noting that, during multicenter validation, efforts should be made to have some geographical diversity among the centers. For example, a three-center validation could be distributed across East, South, and North China. At the same time, attention should be paid to factors such as the race and dietary habits of the recruited population. Lastly, revalidating the diagnostic model’s accuracy in prospective samples is imperative, along with recommended validation in real-world populations (Phase IV). Nevertheless, validation in real-world populations frequently poses challenges, including population diversity, analysis of confounding factors, and financial support.

Cancer initiation and progression are regulated by a combination of genetic and epigenetic events. The complexity of carcinogenesis extends beyond genetic mutations alone and encompass epigenetic modifications as well (Kanwal and Gupta, 2012). Epigenetics is formally characterized as heritable alterations in gene expression or chromosomal stability through mechanisms such as DNA methylation, histone modifications, or non-coding RNAs (e.g., miRNA) without a change in DNA sequence (Ilango et al., 2020). Therefore, in addition to DNA methylation markers, there are currently several other epigenetic and protein markers being explored for the early detection of EC. These include traditional blood tumor markers (such as CEA, Cyfra21-1, p53, SCC-Ag and VEGF-C) (Zhang et al., 2015), DNA fragments (Tomita et al., 2007), mRNA (Kashyap et al., 2009), and miRNA (Xue et al., 2024). However, most marker studies currently lack in-depth investigation and are deficient in repetitive validation. Studies on DNA methylation and miRNA are the most extensive. For example, Jinsei et, al developed an 8-miRNA panel for early detection of ESCC, and verified in multiple cohorts with AUC values of 0.80–0.93 (Miyoshi et al., 2022). Kazuki et, al developed a 6-miRNA panel with sensitivity and specificity of 96% and 98% (Sudo et al., 2019). Although the above studies demonstrate that miRNA has good sensitivity and specificity for diagnosing EC, the short length of miRNA fragments (only 19–24 nt) makes it difficult to distinguish from other similar sequences during detection, resulting in poorer specificity. In contrast, DNA methylation has a significant advantage over miRNA in terms of better specificity. Therefore, in future research on early diagnosis of EC, integrating the various advantages of miRNA and DNA methylation to develop a combined diagnostic kit might be a more promising direction.

## 7 Conclusion

In conclusion, DNA methylation detection holds significant potential as an early detection and screening technology for EC. Among the various approaches, blood cfDNA methylation-based method and esophageal exfoliated cells-based DNA methylation analysis have emerged as two highly promising strategies for early EC detection. The high throughput, high compliance for blood cfDNA methylation-based method and the high sensitivity and specificity of esophageal exfoliated cells-based DNA methylation analysis provide more alternative options for current early detection of EC. However, despite the anticipation of developing numerous methylation markers into commercial kits, there is still a need to enhance their detection sensitivity and specificity. Additionally, standardized pre-analytical procedures are crucial in improving detection performance. We hope that this review serves as an inspirational resource for readers interested in methylated markers for early EC detection, and we anticipate the discovery and validation of an increasing number of methylated markers for clinical testing in EC in the future.

## Abbreviations

5-mC, 5-methylcytosine (5-mC); Dnmt, DNA methyltransferase; AUC, area under the curve; FFT, fresh frozen tissue; FFPE, formalin-fixed and parrffin-embedded; HGD, high-grade dysplasia; LGD, low-grade dysplasia; SQ, normal squamous epithelium; BE, Barrett’s esophagus; GERD, chronic gastroesophageal reflux disease; GC, gastric cancer; CRC, colorectal cancer; EC, esophageal cancer; HCC, hepatocellular carcinoma; LC, lung cancer; PDAC, pancreatic adenocarcinoma; EJC, esophagogastric junction cancer; ESCC, esophageal squamous cell carcinoma; EAC, esophageal adenocarcinoma; OC, ovary cancer; MSP, methylation specific PCR; qMSP, quantitative methylation specific PCR; ddPCR, droplet digital PCR; NGS, Next-Generation Sequencing; TELQAS, target enrichment long-probe quantitative amplified signal; ctDNA, circulating tumor DNA; cfDNA, cell-free DNA; FDA, Food and Drug Administration; NMPA, Chinese National Medical Products Administration.

## References

[B1] Adams L. Roth M. J. Abnet C. C. Dawsey S. P. Qiao Y. L. Wang G. Q. (2008). Promoter methylation in cytology specimens as an early detection marker for esophageal squamous dysplasia and early esophageal squamous cell carcinoma. Cancer Prev. Res. Phila. Pa 1 (5), 357–361. 10.1158/1940-6207.CAPR-08-0061 PMC2615136 19137073

[B2] Akiyama J. Komanduri S. Konda V. J. Mashimo H. Noria S. Triadafilopoulos G. (2014). Endoscopy for diagnosis and treatment in esophageal cancers: high-technology assessment. Ann. N. Y. Acad. Sci. 1325, 77–88. 10.1111/nyas.12526 25266017

[B3] Bian Y. Gao Y. Lu C. Tian B. Xin L. Lin H. (2023). Genome-wide methylation profiling identified methylated KCNA3 and OTOP2 as promising diagnostic markers for esophageal squamous cell carcinoma. Chin. Med. J. 10.1097/CM9.0000000000002832 PMC11268817 37650127

[B4] Bu Q. Luo X. He L. Ma J. He S. Lei W. (2023). Septin9 DNA methylation as a promising biomarker for cervical cancer. J. obstetrics Gynaecol. J. Inst. Obstetrics Gynaecol. 43 (1), 2151356. 10.1080/01443615.2022.2151356 36476308

[B5] Buxbaum J. L. Eloubeidi M. A. (2009). Endoscopic evaluation and treatment of esophageal cance. Minerva Gastroenterol. Dietol. 55 (4), 455–469. 19942829

[B6] Cancer Genome Atlas Research NetworkAnalysis Working Group: Asan UniversityBC Cancer AgencyBrigham and Women’s HospitalBroad InstituteBrown University (2017). Integrated genomic characterization of oesophageal carcinoma. Nature 541 (7636), 169–175. 10.1038/nature20805 28052061 PMC5651175

[B7] Cao C. Q. Chang L. Wu Q. (2020). Circulating methylated Septin 9 and ring finger protein 180 for noninvasive diagnosis of early gastric cancer. Transl. cancer Res. 9 (11), 7012–7021. 10.21037/tcr-20-1330 35117307 PMC8799148

[B8] Chen X. Gole J. Gore A. He Q. Lu M. Min J. (2020). Non-invasive early detection of cancer four years before conventional diagnosis using a blood test. Nat. Commun. 11 (1), 3475. 10.1038/s41467-020-17316-z 32694610 PMC7374162

[B9] Chettouh H. Mowforth O. Galeano-Dalmau N. Bezawada N. Ross-Innes C. MacRae S. (2018). Methylation panel is a diagnostic biomarker for Barrett's oesophagus in endoscopic biopsies and non-endoscopic cytology specimens. Gut 67 (11), 1942–1949. 10.1136/gutjnl-2017-314026 29084829 PMC6176521

[B10] Codipilly D. C. Qin Y. Dawsey S. M. Kisiel J. Topazian M. Ahlquist D. (2018). Screening for esophageal squamous cell carcinoma: recent advances. Gastrointest. Endosc. 88 (3), 413–426. 10.1016/j.gie.2018.04.2352 29709526 PMC7493990

[B11] Dai Y. Li H. Wu Q. Wang J. Wang K. Fei S. (2023). A sensitive and robust plasma-based DNA methylation panel for early detection of target gastrointestinal cancers. Neoplasia (New York, NY) 46, 100941. 10.1016/j.neo.2023.100941 PMC10643353 37918207

[B12] Dan Lister A. F. Maheshwari S. Bradley P. S. Lee V. T. deGuzman B. J. Verma S. (2023). Clinical utility of EsoGuard® on samples collected with EsoCheck® as a triage to endoscopy for identification of barrett’s esophagus – interim data from the CLUE study. Archives Clin. Biomed. Res. 7, 626–634. 10.26502/acbr.50170378

[B13] Das M. Sharma S. K. Sekhon G. S. Saikia B. J. Mahanta J. Phukan R. K. (2014). Promoter methylation of MGMT gene in serum of patients with esophageal squamous cell carcinoma in North East India. Asian Pac. J. cancer Prev. APJCP 15 (22), 9955–9960. 10.7314/apjcp.2014.15.22.9955 25520135

[B14] De Mattos-Arruda L. Mayor R. Ng C. K. Y. Weigelt B. Martínez-Ricarte F. Torrejon D. (2015). Cerebrospinal fluid-derived circulating tumour DNA better represents the genomic alterations of brain tumours than plasma. Nat. Commun. 6, 8839. 10.1038/ncomms9839 26554728 PMC5426516

[B15] Dong S. W. Zhang P. Liu Y. M. Cui Y. T. Wang S. Liang S. J. (2012). Study on RIZ1 gene promoter methylation status in human esophageal squamous cell carcinoma. World J. gastroenterology 18 (6), 576–582. 10.3748/wjg.v18.i6.576 PMC3280405 22363126

[B16] Duffy M. J. Diamandis E. P. Crown J. (2021). Circulating tumor DNA (ctDNA) as a pan-cancer screening test: is it finally on the horizon? Clin. Chem. laboratory Med. 59 (8), 1353–1361. 10.1515/cclm-2021-0171 33856748

[B17] Evans J. A. Early D. S. Chandraskhara V. Chathadi K. V. Fanelli R. D. Fisher D. A. (2013). The role of endoscopy in the assessment and treatment of esophageal cancer. Gastrointest. Endosc. 77 (3), 328–334. 10.1016/j.gie.2012.10.001 23410694

[B18] Fan Z. Qin Y. Zhou J. Chen R. Gu J. Li M. (2022). Feasibility of using P16 methylation as a cytologic marker for esophageal squamous cell carcinoma screening: a pilot study. Cancer Med. 11 (21), 4033–4042. 10.1002/cam4.4718 35352503 PMC9636512

[B19] Fitzgerald R. C. di Pietro M. O'Donovan M. Maroni R. Muldrew B. Debiram-Beecham I. (2020). Cytosponge-trefoil factor 3 versus usual care to identify Barrett's oesophagus in a primary care setting: a multicentre, pragmatic, randomised controlled trial. Lancet London, Engl. 396 (10247), 333–344. 10.1016/S0140-6736(20)31099-0 PMC7408501 32738955

[B20] Gao Q. Lin Y. P. Li B. S. Wang G. Q. Dong L. Q. Shen B. Y. (2023). Unintrusive multi-cancer detection by circulating cell-free DNA methylation sequencing (THUNDER): development and independent validation studies. Ann. Oncol. official J. Eur. Soc. Med. Oncol. 34 (5), 486–495. 10.1016/j.annonc.2023.02.010 36849097

[B21] Hamilton J. P. Sato F. Jin Z. Greenwald B. D. Ito T. Mori Y. (2006). Reprimo methylation is a potential biomarker of Barrett's-Associated esophageal neoplastic progression. Clin. cancer Res. official J. Am. Assoc. Cancer Res. 12 (22), 6637–6642. 10.1158/1078-0432.CCR-06-1781 17121882

[B22] Henry M. A. Lerco M. M. Ribeiro P. W. Rodrigues M. A. (2014). Epidemiological features of esophageal cancer. Squamous cell carcinoma versus adenocarcinoma. Acta cir. bras. 29 (6), 389–393. 10.1590/s0102-86502014000600007 24919048

[B23] Hibi K. Goto T. Shirahata A. Saito M. Kigawa G. Nemoto H. (2011). Detection of TFPI2 methylation in the serum of colorectal cancer patients. Cancer Lett. 311 (1), 96–100. 10.1016/j.canlet.2011.07.006 21820798

[B24] Hibi K. Taguchi M. Nakayama H. Takase T. Kasai Y. Ito K. (2001). Molecular detection of p16 promoter methylation in the serum of patients with esophageal squamous cell carcinoma. Clin. cancer Res. official J. Am. Assoc. Cancer Res. 7 (10), 3135–3138. 11595706

[B25] Hidestrand M. Stokowski R. Song K. Oliphant A. Deavers J. Goetsch M. (2012). Influence of temperature during transportation on cell-free DNA analysis. Fetal diagnosis Ther. 31 (2), 122–128. 10.1159/000335020 22261730

[B26] Hou P. Ji M. J. Shen J. Y. He N. Y. Lu Z. H. (2005). Detection of p16 hypermethylation in gastric carcinomas using a seminested methylation-specific PCR. Biochem. Genet. 43 (1-2), 1–9. 10.1007/s10528-005-1062-8 15859515

[B27] Huang J. Wang G. Tang J. Zhuang W. Wang L. P. Liou Y. L. (2017). DNA methylation status of PAX1 and ZNF582 in esophageal squamous cell carcinoma. Int. J. Environ. Res. public health 14 (2), 216. 10.3390/ijerph14020216 28241446 PMC5334770

[B28] Husain H. Melnikova V. O. Kosco K. Woodward B. More S. Pingle S. C. (2017). Monitoring daily dynamics of early tumor response to targeted therapy by detecting circulating tumor DNA in urine. Clin. cancer Res. official J. Am. Assoc. Cancer Res. 23 (16), 4716–4723. 10.1158/1078-0432.CCR-17-0454 PMC5737735 28420725

[B29] Ikoma D. Ichikawa D. Ueda Y. Tani N. Tomita H. Sai S. (2007). Circulating tumor cells and aberrant methylation as tumor markers in patients with esophageal cancer. Anticancer Res. 27 (1b), 535–539. 17348438

[B30] Ilango S. Paital B. Jayachandran P. Padma P. R. Nirmaladevi R. (2020). Epigenetic alterations in cancer. cancer 25 (6), 1058–1109. 10.2741/4847 32114424

[B31] Imperiale T. F. Ransohoff D. F. Itzkowitz S. H. Levin T. R. Lavin P. Lidgard G. P. (2014). Multitarget stool DNA testing for colorectal-cancer screening. N. Engl. J. Med. 370 (14), 1287–1297. 10.1056/NEJMoa1311194 24645800

[B32] Irizarry R. A. Ladd-Acosta C. Wen B. Wu Z. Montano C. Onyango P. (2009a). The human colon cancer methylome shows similar hypo- and hypermethylation at conserved tissue-specific CpG island shores. Nat. Genet. 41 (2), 178–186. 10.1038/ng.298 19151715 PMC2729128

[B33] Irizarry R. A. Wu H. Feinberg A. P. (2009b). A species-generalized probabilistic model-based definition of CpG islands. Mammalian genome official J. Int. Mammalian Genome Soc. 20 (9-10), 674–680. 10.1007/s00335-009-9222-5 PMC2962567 19777308

[B34] Iyer P. G. Taylor W. R. Johnson M. L. Lansing R. L. Maixner K. A. Hemminger L. L. (2020). Accurate nonendoscopic detection of Barrett's esophagus by methylated DNA markers: a multisite case control study. Am. J. gastroenterology 115 (8), 1201–1209. 10.14309/ajg.0000000000000656 PMC7415629 32558685

[B35] Iyer P. G. Taylor W. R. Johnson M. L. Lansing R. L. Maixner K. A. Yab T. C. (2018). Highly discriminant methylated DNA markers for the non-endoscopic detection of Barrett's esophagus. Am. J. gastroenterology 113 (8), 1156–1166. 10.1038/s41395-018-0107-7 29891853

[B36] Iyer P. G. Taylor W. R. Slettedahl S. W. Lansing R. L. Hemminger L. L. Cayer F. K. (2021). Validation of a methylated DNA marker panel for the nonendoscopic detection of Barrett's esophagus in a multisite case-control study. Gastrointest. Endosc. 94 (3), 498–505. 10.1016/j.gie.2021.03.937 33857451 PMC8380660

[B37] Jamshidi A. Liu M. C. Klein E. A. Venn O. Hubbell E. Beausang J. F. (2022). Evaluation of cell-free DNA approaches for multi-cancer early detection. Cancer Cell 40 (12), 1537–1549.e12. 10.1016/j.ccell.2022.10.022 36400018

[B38] Januszewicz W. Tan W. K. Lehovsky K. Debiram-Beecham I. Nuckcheddy T. Moist S. (2019). Safety and acceptability of esophageal Cytosponge cell collection device in a pooled analysis of data from individual patients. Clin. gastroenterology hepatology official Clin. Pract. J. Am. Gastroenterological Assoc. 17 (4), 647–656.e1. 10.1016/j.cgh.2018.07.043 PMC6370042 30099104

[B39] Jia Y. Yang Y. Brock M. V. Cao B. Zhan Q. Li Y. (2012). Methylation of TFPI-2 is an early event of esophageal carcinogenesis. Epigenomics 4 (2), 135–146. 10.2217/epi.12.11 22449186 PMC3742137

[B40] Jin Z. Olaru A. Yang J. Sato F. Cheng Y. Kan T. (2007). Hypermethylation of tachykinin-1 is a potential biomarker in human esophageal cancer. Clin. cancer Res. official J. Am. Assoc. Cancer Res. 13 (21), 6293–6300. 10.1158/1078-0432.CCR-07-0818 17975140

[B41] Kadri S. R. Lao-Sirieix P. O'Donovan M. Debiram I. Das M. Blazeby J. M. (2010). Acceptability and accuracy of a non-endoscopic screening test for Barrett's oesophagus in primary care: cohort study. BMJ Clin. Res. ed 341, c4372. 10.1136/bmj.c4372 PMC2938899 20833740

[B42] Kandimalla R. Xu J. Link A. Matsuyama T. Yamamura K. Parker M. I. (2021). EpiPanGI dx: a cell-free DNA methylation fingerprint for the early detection of gastrointestinal cancers. Clin. cancer Res. official J. Am. Assoc. Cancer Res. 27 (22), 6135–6144. 10.1158/1078-0432.CCR-21-1982 PMC8595812 34465601

[B43] Kanwal R. Gupta S. (2012). Epigenetic modifications in cancer. cancer 81 (4), 303–311. 10.1111/j.1399-0004.2011.01809.x PMC3590802 22082348

[B44] Kashyap M. K. Marimuthu A. Kishore C. J. Peri S. Keerthikumar S. Prasad T. S. (2009). Genomewide mRNA profiling of esophageal squamous cell carcinoma for identification of cancer biomarkers. Cancer Biol. Ther. 8 (1), 36–46. 10.4161/cbt.8.1.7090 18981721

[B45] Kaz A. M. Luo Y. Dzieciatkowski S. Chak A. Willis J. E. Upton M. P. (2012). Aberrantly methylated PKP1 in the progression of Barrett's esophagus to esophageal adenocarcinoma. Genes, chromosomes cancer. 51 (4), 384–393. 10.1002/gcc.21923 22170739 PMC3292431

[B46] Kerachian M. A. Azghandi M. Mozaffari-Jovin S. Thierry A. R. (2021). Guidelines for pre-analytical conditions for assessing the methylation of circulating cell-free DNA. Clin. epigenetics 13 (1), 193. 10.1186/s13148-021-01182-7 34663458 PMC8525023

[B47] Klein E. A. Richards D. Cohn A. Tummala M. Lapham R. Cosgrove D. (2021). Clinical validation of a targeted methylation-based multi-cancer early detection test using an independent validation set. Ann. Oncol. official J. Eur. Soc. Med. Oncol. 32 (9), 1167–1177. 10.1016/j.annonc.2021.05.806 34176681

[B48] Lao-Sirieix P. Boussioutas A. Kadri S. R. O'Donovan M. Debiram I. Das M. (2009). Non-endoscopic screening biomarkers for Barrett's oesophagus: from microarray analysis to the clinic. Gut 58 (11), 1451–1459. 10.1136/gut.2009.180281 19651633

[B49] Lee J. S. Kim M. Seong M. W. Kim H. S. Lee Y. K. Kang H. J. (2020). Plasma vs. serum in circulating tumor DNA measurement: characterization by DNA fragment sizing and digital droplet polymerase chain reaction. Clin. Chem. laboratory Med. 58 (4), 527–532. 10.1515/cclm-2019-0896 31874093

[B50] Levine M. S. Rubesin S. E. (2017). History and evolution of the barium swallow for evaluation of the pharynx and esophagus. Dysphagia 32 (1), 55–72. 10.1007/s00455-016-9774-y 28101664

[B51] Li B. Wang B. Niu L. J. Jiang L. Qiu C. C. (2011). Hypermethylation of multiple tumor-related genes associated with DNMT3b up-regulation served as a biomarker for early diagnosis of esophageal squamous cell carcinoma. Epigenetics 6 (3), 307–316. 10.4161/epi.6.3.14182 21150312 PMC3092679

[B52] Li N. He Y. Mi P. Hu Y. (2019). ZNF582 methylation as a potential biomarker to predict cervical intraepithelial neoplasia type III/worse: a meta-analysis of related studies in Chinese population. Medicine 98 (6), e14297. 10.1097/MD.0000000000014297 30732145 PMC6380660

[B53] Li X. Zhou F. Jiang C. Wang Y. Lu Y. Yang F. (2014). Identification of a DNA methylome profile of esophageal squamous cell carcinoma and potential plasma epigenetic biomarkers for early diagnosis. PloS one 9 (7), e103162. 10.1371/journal.pone.0103162 25050929 PMC4106874

[B54] Liu C. Li N. Lu H. Wang Z. Chen C. Wu L. (2015). Circulating SFRP1 promoter methylation status in gastric adenocarcinoma and esophageal square cell carcinoma. Biomed. Rep. 3 (1), 123–127. 10.3892/br.2014.388 25469261 PMC4251162

[B55] Liu M. C. Oxnard G. R. Klein E. A. Swanton C. Seiden M. V. CCGA Consortium (2020b). Sensitive and specific multi-cancer detection and localization using methylation signatures in cell-free DNA. Ann. Oncol. official J. Eur. Soc. Med. Oncol. 31 (6), 745–759. 10.1016/j.annonc.2020.02.011 PMC8274402 33506766

[B56] Liu Y. Zhao G. Miao J. Li H. Ma Y. Liu X. (2020a). Performance comparison between plasma and stool methylated SEPT9 tests for detecting colorectal cancer. Cancer. Front. Genet. 11, 324. 10.3389/fgene.2020.00324 32373158 PMC7176978

[B57] Lou-Qian Z. Rong Y. Ming L. Xin Y. Feng J. Lin X. (2013). The prognostic value of epigenetic silencing of p16 gene in NSCLC patients: a systematic review and meta-analysis. PloS one 8 (1), e54970. 10.1371/journal.pone.0054970 23372805 PMC3555860

[B58] Luo H. Zhao Q. Wei W. Zheng L. Yi S. Li G. (2020). Circulating tumor DNA methylation profiles enable early diagnosis, prognosis prediction, and screening for colorectal cancer. Sci. Transl. Med. 12 (524), eaax7533. 10.1126/scitranslmed.aax7533 31894106

[B59] Ma K. Kalra A. Tsai H. L. Okello S. Cheng Y. Meltzer S. J. (2022). Accurate nonendoscopic detection of esophageal squamous cell carcinoma using methylated DNA biomarkers. Gastroenterology 163 (2), 507–509.e2. 10.1053/j.gastro.2022.04.021 35483446 PMC9555873

[B60] Mao R. Chou L. S. (2010). Methylation analysis by restriction endonuclease digestion and real-time PCR. Clin. Chem. 56 (7), 1050–1052. 10.1373/clinchem.2010.146654 20472820

[B61] Meng Y. Wang Q.-G. Wang J.-X. Zhu S.-T. Jiao Y. Li P. (2011). Epigenetic inactivation of the SFRP1 gene in esophageal squamous cell carcinoma. Dig. Dis. Sci. 56 (11), 3195–3203. 10.1007/s10620-011-1734-7 21567192

[B62] Miyoshi J. Zhu Z. Luo A. Toden S. Zhou X. Izumi D. (2022). A microRNA-based liquid biopsy signature for the early detection of esophageal squamous cell carcinoma: a retrospective, prospective and multicenter study. Mol. cancer 21 (1), 44. 10.1186/s12943-022-01507-x 35148754 PMC8832722

[B63] Moinova H. R. LaFramboise T. Lutterbaugh J. D. Chandar A. K. Dumot J. Faulx A. (2018). Identifying DNA methylation biomarkers for non-endoscopic detection of Barrett's esophagus. Sci. Transl. Med. 10 (424), eaao5848. 10.1126/scitranslmed.aao5848 29343623 PMC5789768

[B64] Oussalah A. Rischer S. Bensenane M. Conroy G. Filhine-Tresarrieu P. Debard R. (2018). Plasma mSEPT9: a novel circulating cell-free DNA-based epigenetic biomarker to diagnose hepatocellular carcinoma. EBioMedicine 30, 138–147. 10.1016/j.ebiom.2018.03.029 29627389 PMC5952996

[B65] Paterson A. L. Gehrung M. Fitzgerald R. C. O'Donovan M. (2020). Role of TFF3 as an adjunct in the diagnosis of Barrett's esophagus using a minimally invasive esophageal sampling device-The Cytosponge(TM). Diagn. Cytopathol. 48 (3), 253–264. 10.1002/dc.24354 31814330 PMC7075710

[B66] Pei B. Zhao G. Geng Z. Wang Y. Wang M. Wang X. (2023). Identifying potential DNA methylation markers for the detection of esophageal cancer in plasma. Front. Genet. 14, 1222617. 10.3389/fgene.2023.1222617 37867599 PMC10586502

[B67] Peng C. Zhao G. Pei B. Wang K. Li H. Fei S. (2022). A novel plasma-based methylation panel for upper gastrointestinal cancer early detection. Cancers 14 (21), 5282. 10.3390/cancers14215282 36358701 PMC9656240

[B68] Pittella-Silva F. Chin Y. M. Chan H. T. Nagayama S. Miyauchi E. Low S. K. (2020). Plasma or serum: which is preferable for mutation detection in liquid biopsy? Clin. Chem. 66 (7), 946–957. 10.1093/clinchem/hvaa103 32516802

[B69] Potter N. T. Hurban P. White M. N. Whitlock K. D. Lofton-Day C. E. Tetzner R. (2014). Validation of a real-time PCR-based qualitative assay for the detection of methylated SEPT9 DNA in human plasma. Clin. Chem. 60 (9), 1183–1191. 10.1373/clinchem.2013.221044 24938752

[B70] Prasoppokakorn T. Buntho A. Ingrungruanglert P. Tiyarattanachai T. Jaihan T. Kulkraisri K. (2022). Circulating tumor cells as a prognostic biomarker in patients with hepatocellular carcinoma. Sci. Rep. 12 (1), 18686. 10.1038/s41598-022-21888-9 36333384 PMC9636215

[B71] Pu W. Wang C. Chen S. Zhao D. Zhou Y. Ma Y. (2017). Targeted bisulfite sequencing identified a panel of DNA methylation-based biomarkers for esophageal squamous cell carcinoma (ESCC). Clin. epigenetics 9, 129. 10.1186/s13148-017-0430-7 29270239 PMC5732523

[B72] Qiao G. Zhuang W. Dong B. Li C. Xu J. Wang G. (2021). Discovery and validation of methylation signatures in circulating cell-free DNA for early detection of esophageal cancer: a case-control study. BMC Med. 19 (1), 243. 10.1186/s12916-021-02109-y 34641873 PMC8513367

[B73] Qin Y. Wu C. W. Taylor W. R. Sawas T. Burger K. N. Mahoney D. W. (2019). Discovery, validation, and application of novel methylated DNA markers for detection of esophageal cancer in plasma. Clin. cancer Res. official J. Am. Assoc. Cancer Res. 25 (24), 7396–7404. 10.1158/1078-0432.CCR-19-0740 PMC6911634 31527170

[B74] Rahat B. Ali T. Sapehia D. Mahajan A. Kaur J. (2020). Circulating cell-free nucleic acids as epigenetic biomarkers in precision medicine. Front. Genet. 11, 844. 10.3389/fgene.2020.00844 32849827 PMC7431953

[B75] Ramalho-Carvalho J. Henrique R. Jerónimo C. (2018). “Methylation-specific PCR,” in DNA methylation protocols. Editor Tost J. (New York, NY: Springer New York), 447–472. 10.1007/978-1-4939-7481-8_23 29224158

[B76] Rasmussen S. L. Krarup H. B. Sunesen K. G. Pedersen I. S. Madsen P. H. Thorlacius-Ussing O. (2016). Hypermethylated DNA as a biomarker for colorectal cancer: a systematic review. Colorectal Dis. official J. Assoc. Coloproctology G. B. Irel. 18 (6), 549–561. 10.1111/codi.13336 26998585

[B77] Reeh M. Effenberger K. E. Koenig A. M. Riethdorf S. Eichstädt D. Vettorazzi E. (2015). Circulating tumor cells as a biomarker for preoperative prognostic staging in patients with esophageal cancer. Ann. Surg. 261 (6), 1124–1130. 10.1097/SLA.0000000000001130 25607767

[B78] Rogers J. E. Ajani J. A. (2022). Perspectives on the pharmacological management of esophageal cancer: where are we now and where do we need to go? Expert Opin. Pharmacother. 23 (17), 1893–1902. 10.1080/14656566.2022.2140585 36286544

[B79] Rokni P. Shariatpanahi A. M. Sakhinia E. Kerachian M. A. (2018). BMP3 promoter hypermethylation in plasma-derived cell-free DNA in colorectal cancer patients. Genes & genomics 40 (4), 423–428. 10.1007/s13258-017-0644-2 29892846

[B80] Roth M. J. Abnet C. C. Hu N. Wang Q. H. Wei W. Q. Green L. (2006). p16, MGMT, RARβ2, CLDN3, CRBP and MT1G gene methylation in esophageal squamous cell carcinoma and its precursor lesions. Oncol. Rep. 15 (6), 1591–1597. 10.3892/or.15.6.1591 16685400

[B81] Rothwell D. G. Ayub M. Cook N. Thistlethwaite F. Carter L. Dean E. (2019). Utility of ctDNA to support patient selection for early phase clinical trials: the TARGET study. Nat. Med. 25 (5), 738–743. 10.1038/s41591-019-0380-z 31011204

[B82] Ruddon MDPDRW (2010). Chapter 1 - introduction to the molecular biology of cancer: translation to the clinic. Prog. Mol. Biol. Transl. Sci. 95, 1–8. 21075326 10.1016/B978-0-12-385071-3.00001-0

[B83] Salta S. Macedo-Silva C. Miranda-Gonçalves V. Lopes N. Gigliano D. Guimarães R. (2020). A DNA methylation-based test for esophageal cancer detection. Biomark. Res. 8 (1), 68. 10.1186/s40364-020-00248-7 33292587 PMC7691099

[B84] Schlick K. Markus S. Huemer F. Ratzinger L. Zaborsky N. Clemens H. (2021). Evaluation of circulating cell-free KRAS mutational status as a molecular monitoring tool in patients with pancreatic cancer. Pancreatol. official J. Int. Assoc. Pancreatol. (IAP) 21 (8), 1466–1471. 10.1016/j.pan.2021.09.004 34511398

[B85] Shaheen N. J. Falk G. W. Iyer P. G. Gerson L. B. American College of Gastroenterology (2016). ACG clinical guideline: diagnosis and management of Barrett's esophagus. Am. J. gastroenterology 111 (1), 30–51. quiz 1. 10.1038/ajg.2015.322 PMC10245082 26526079

[B86] Shahsavari D. Kudaravalli P. Yap J. E. L. Vega K. J. (2022). Expanding beyond endoscopy: a review of non-invasive modalities in Barrett's esophagus screening and surveillance. World J. gastroenterology 28 (32), 4516–4526. 10.3748/wjg.v28.i32.4516 PMC9476875 36157931

[B87] Siegel R. L. Miller K. D. Fuchs H. E. Jemal A. (2021). Cancer statistics, 2021. CA a cancer J. Clin. 71 (1), 7–33. 10.3322/caac.21654 33433946

[B88] Siegfried Z. Simon I. (2010). DNA methylation and gene expression. Wiley Interdiscip. Rev. Syst. Biol. Med. 2 (3), 362–371. 10.1002/wsbm.64 20836034

[B89] Sigalotti L. Covre A. Colizzi F. Fratta E. (2019). Quantitative methylation-specific PCR: a simple method for studying epigenetic modifications of cell-free DNA. Methods Mol. Biol. Clift. NJ 1909, 137–162. 10.1007/978-1-4939-8973-7_11 30580429

[B90] Song L. Chen Y. Gong Y. Wan J. Guo S. Liu H. (2020). Opportunistic screening and survival prediction of digestive cancers by the combination of blood mSEPT9 with protein markers. Ther. Adv. Med. Oncol. 12, 1758835920962966. 10.1177/1758835920962966 33403008 PMC7745555

[B91] Song L. Li Y. (2015). SEPT9: a specific circulating biomarker for colorectal cancer. Adv. Clin. Chem. 72, 171–204. 10.1016/bs.acc.2015.07.004 26471083

[B92] Sorber L. Zwaenepoel K. Deschoolmeester V. Roeyen G. Lardon F. Rolfo C. (2017). A comparison of cell-free DNA isolation kits: isolation and quantification of cell-free DNA in plasma. J. Mol. diagnostics JMD 19 (1), 162–168. 10.1016/j.jmoldx.2016.09.009 27865784

[B93] Sudo K. Kato K. Matsuzaki J. Boku N. Abe S. Saito Y. (2019). Development and validation of an esophageal squamous cell carcinoma detection model by large-scale MicroRNA profiling. JAMA Netw. open 2 (5), e194573. 10.1001/jamanetworkopen.2019.4573 31125107 PMC6632131

[B94] Sung H. Ferlay J. Siegel R. L. Laversanne M. Soerjomataram I. Jemal A. (2021). Global cancer statistics 2020: GLOBOCAN estimates of incidence and mortality worldwide for 36 cancers in 185 countries. CA a cancer J. Clin. 71 (3), 209–249. 10.3322/caac.21660 33538338

[B95] Talukdar F. R. di Pietro M. Secrier M. Moehler M. Goepfert K. Lima S. S. C. (2018). Molecular landscape of esophageal cancer: implications for early detection and personalized therapy. Ann. N. Y. Acad. Sci. 1434 (1), 342–359. 10.1111/nyas.13876 29917250

[B96] Talukdar F. R. Soares Lima S. C. Khoueiry R. Laskar R. S. Cuenin C. Sorroche B. P. (2021). Genome-wide DNA methylation profiling of esophageal squamous cell carcinoma from global high-incidence regions identifies crucial genes and potential cancer markers. Cancer Res. 81 (10), 2612–2624. 10.1158/0008-5472.CAN-20-3445 33741694

[B97] Tang L. Liou Y. L. Wan Z. R. Tang J. Zhou Y. Zhuang W. (2019). Aberrant DNA methylation of PAX1, SOX1 and ZNF582 genes as potential biomarkers for esophageal squamous cell carcinoma. Biomed. Pharmacother. = Biomedecine Pharmacother. 120, 109488. 10.1016/j.biopha.2019.109488 31629253

[B98] Thierry A. R. El Messaoudi S. Gahan P. B. Anker P. Stroun M. (2016). Origins, structures, and functions of circulating DNA in oncology. Cancer metastasis Rev. 35 (3), 347–376. 10.1007/s10555-016-9629-x 27392603 PMC5035665

[B99] Tomita H. Ichikawa D. Ikoma D. Sai S. Tani N. Ikoma H. (2007). Quantification of circulating plasma DNA fragments as tumor markers in patients with esophageal cancer. Anticancer Res. 27 (4c), 2737–2741. 17695440

[B100] van Eijk K. R. de Jong S. Boks M. P. Langeveld T. Colas F. Veldink J. H. (2012). Genetic analysis of DNA methylation and gene expression levels in whole blood of healthy human subjects. BMC genomics 13, 636. 10.1186/1471-2164-13-636 23157493 PMC3583143

[B101] Váraljai R. Wistuba-Hamprecht K. Seremet T. Diaz J. M. S. Nsengimana J. Sucker A. (2020). Application of circulating cell-free tumor DNA profiles for therapeutic monitoring and outcome prediction in genetically heterogeneous metastatic melanoma. JCO Precis. Oncol. 3, 1–10. 10.1200/PO.18.00229 PMC7446476 32914028

[B102] Wang C. Pu W. Zhao D. Zhou Y. Lu T. Chen S. (2018a). Identification of hyper-methylated tumor suppressor genes-based diagnostic panel for esophageal squamous cell carcinoma (ESCC) in a Chinese han population. Front. Genet. 9, 356. 10.3389/fgene.2018.00356 30233644 PMC6133993

[B103] Wang H. DeFina S. M. Bajpai M. Yan Q. Yang L. Zhou Z. (2021). DNA methylation markers in esophageal cancer: an emerging tool for cancer surveillance and treatment. Am. J. cancer Res. 11 (11), 5644–5658. 34873485 PMC8640794

[B104] Wang H. Q. Yang C. Y. Wang S. Y. Wang T. Han J. L. Wei K. (2018b). Cell-free plasma hypermethylated CASZ1, CDH13 and ING2 are promising biomarkers of esophageal cancer. J. Biomed. Res. 32 (5), 424–433. 10.7555/JBR.32.20170065 30355852 PMC6283827

[B105] Wang J. Liu S. Wang H. Zheng L. Zhou C. Li G. (2020). Robust performance of a novel stool DNA test of methylated SDC2 for colorectal cancer detection: a multicenter clinical study. Clin. epigenetics 12 (1), 162. 10.1186/s13148-020-00954-x 33126908 PMC7602331

[B106] Wang J. Sasco A. J. Fu C. Xue H. Guo G. Hua Z. (2008). Aberrant DNA methylation of P16, MGMT, and hMLH1 genes in combination with MTHFR C677T genetic polymorphism in esophageal squamous cell carcinoma. Cancer Epidemiol. biomarkers Prev. a Publ. Am. Assoc. Cancer Res. cosponsored by Am. Soc. Prev. Oncol. 17 (1), 118–125. 10.1158/1055-9965.EPI-07-0733 18199718

[B107] Wang Y. Springer S. Mulvey C. L. Silliman N. Schaefer J. Sausen M. (2015). Detection of somatic mutations and HPV in the saliva and plasma of patients with head and neck squamous cell carcinomas. Sci. Transl. Med. 7 (293), 293ra104. 10.1126/scitranslmed.aaa8507 PMC4587492 26109104

[B108] Wang Z. Kambhampati S. Cheng Y. Ma K. Simsek C. Tieu A. H. (2019). Methylation biomarker panel performance in EsophaCap cytology samples for diagnosing Barrett's esophagus: a prospective validation study. Clin. cancer Res. official J. Am. Assoc. Cancer Res. 25 (7), 2127–2135. 10.1158/1078-0432.CCR-18-3696 PMC6594757 30670490

[B109] Worm Ørntoft M. B. Jensen S. Hansen T. B. Bramsen J. B. Andersen C. L. (2017). Comparative analysis of 12 different kits for bisulfite conversion of circulating cell-free DNA. Epigenetics 12 (8), 626–636. 10.1080/15592294.2017.1334024 28557629 PMC5687322

[B110] Wu D. Zhou G. Jin P. Zhu J. Li S. Wu Q. (2016). Detection of colorectal cancer using a simplified SEPT9 gene methylation assay is a reliable method for opportunistic screening. J. Mol. diagnostics JMD 18 (4), 535–545. 10.1016/j.jmoldx.2016.02.005 27133379

[B111] Xi Y. Lin Y. Guo W. Wang X. Zhao H. Miao C. (2022). Multi-omic characterization of genome-wide abnormal DNA methylation reveals diagnostic and prognostic markers for esophageal squamous-cell carcinoma. Signal Transduct. Target. Ther. 7 (1), 53. 10.1038/s41392-022-00873-8 35210398 PMC8873499

[B112] Xue Y. Wang K. Jiang Y. Dai Y. Liu X. Pei B. (2024). An ultrasensitive and multiplexed miRNA one-step real time RT-qPCR detection system and its application in esophageal cancer serum. Biosens. Bioelectron. 247, 115927. 10.1016/j.bios.2023.115927 38113694

[B113] Yamaguchi S. Kato H. Miyazaki T. Sohda M. Kimura H. Ide M. (2005). RASSF1A gene promoter methylation in esophageal cancer specimens. Dis. esophagus official J. Int. Soc. Dis. Esophagus 18 (4), 253–256. 10.1111/j.1442-2050.2005.00501.x 16128782

[B114] Yang C. S. Chen X. L. (2021). Research on esophageal cancer: with personal perspectives from studies in China and Kenya. Int. J. cancer 149 (2), 264–276. 10.1002/ijc.33421 33270917 PMC8141013

[B115] Yang J. Liu X. Cao S. Dong X. Rao S. Cai K. (2020). Understanding esophageal cancer: the challenges and opportunities for the Next decade. Front. Oncol. 10, 1727. 10.3389/fonc.2020.01727 33014854 PMC7511760

[B116] You Y. J. Chen Y. P. Zheng X. X. Meltzer S. J. Zhang H. (2012). Aberrant methylation of the PTPRO gene in peripheral blood as a potential biomarker in esophageal squamous cell carcinoma patients. Cancer Lett. 315 (2), 138–144. 10.1016/j.canlet.2011.08.032 22099875 PMC3248961

[B117] Yu M. Moinova H. R. Willbanks A. Cannon V. K. Wang T. Carter K. (2022). Novel DNA methylation biomarker panel for detection of esophageal adenocarcinoma and high-grade dysplasia. Clin. cancer Res. official J. Am. Assoc. Cancer Res. 28 (17), 3761–3769. 10.1158/1078-0432.CCR-22-0445 PMC9444948 35705525

[B118] Yu M. O'Leary R. M. Kaz A. M. Morris S. M. Carter K. T. Chak A. (2015). Methylated B3GAT2 and ZNF793 are potential detection biomarkers for Barrett's esophagus. Cancer Epidemiol. biomarkers Prev. a Publ. Am. Assoc. Cancer Res. cosponsored by Am. Soc. Prev. Oncol. 24 (12), 1890–1897. 10.1158/1055-9965.EPI-15-0370 PMC4670566 26545406

[B119] Zhang G. He F. Zhao G. Huang Z. Li X. Xia X. (2021). Combining serum DNA methylation biomarkers and protein tumor markers improved clinical sensitivity for early detection of colorectal cancer. Int. J. genomics 2021, 6613987. 10.1155/2021/6613987 33977101 PMC8084680

[B120] Zhang J. Zhu Z. Liu Y. Jin X. Xu Z. Yu Q. (2015). Diagnostic value of multiple tumor markers for patients with esophageal carcinoma. PloS one 10 (2), e0116951. 10.1371/journal.pone.0116951 25693076 PMC4333286

[B121] Zhang L. Yang X. Tian Y. Yu Q. Zhou D. Wu Z. (2022). Noninvasive detection of esophageal cancer by the combination of mSEPT9 and SNCG. Genet. Test. Mol. biomarkers 26 (1), 8–16. 10.1089/gtmb.2021.0089 35089073

[B122] Zhao G. Li H. Yang Z. Wang Z. Xu M. Xiong S. (2019). Multiplex methylated DNA testing in plasma with high sensitivity and specificity for colorectal cancer screening. Cancer Med. 8 (12), 5619–5628. 10.1002/cam4.2475 31407497 PMC6745865

[B123] Zheng R. S. Sun K. X. Zhang S. W. Zeng H. M. Zou X. N. Chen R. (2019). Report of cancer epidemiology in China, 2015. Zhonghua zhong liu za zhi Chin. J. Oncol. 41 (1), 19–28. 10.3760/cma.j.issn.0253-3766.2019.01.005 30678413

[B124] Zhou Z. Kalatskaya I. Russell D. Marcon N. Cirocco M. Krzyzanowski P. M. (2019). Combined EsophaCap cytology and MUC2 immunohistochemistry for screening of intestinal metaplasia, dysplasia and carcinoma. Clin. Exp. gastroenterology 12, 219–229. 10.2147/CEG.S186958 PMC6527096 31190949

[B125] Zou H. Molina J. R. Harrington J. J. Osborn N. K. Klatt K. K. Romero Y. (2005). Aberrant methylation of secreted frizzled-related protein genes in esophageal adenocarcinoma and Barrett's esophagus. Int. J. cancer 116 (4), 584–591. 10.1002/ijc.21045 15825175

[B126] Zou H. Z. Yu B. M. Wang Z. W. Sun J. Y. Cang H. Gao F. (2002). Detection of aberrant p16 methylation in the serum of colorectal cancer patients. Clin. cancer Res. official J. Am. Assoc. Cancer Res. 8 (1), 188–191. 11801557

